# A Roadmap for Integrating Neuroscience Into Addiction Treatment: A Consensus of the Neuroscience Interest Group of the International Society of Addiction Medicine

**DOI:** 10.3389/fpsyt.2019.00877

**Published:** 2019-12-23

**Authors:** Antonio Verdejo-Garcia, Valentina Lorenzetti, Victoria Manning, Hugh Piercy, Raimondo Bruno, Rob Hester, David Pennington, Serenella Tolomeo, Shalini Arunogiri, Marsha E. Bates, Henrietta Bowden-Jones, Salvatore Campanella, Stacey B. Daughters, Christos Kouimtsidis, Dan I. Lubman, Dieter J. Meyerhoff, Annaketurah Ralph, Tara Rezapour, Hosna Tavakoli, Mehran Zare-Bidoky, Anna Zilverstand, Douglas Steele, Scott J. Moeller, Martin Paulus, Alex Baldacchino, Hamed Ekhtiari

**Affiliations:** ^1^Turner Institute for Brain and Mental Health, Monash University, Melbourne, VIC, Australia; ^2^School of Psychology, Faculty of Health Sciences, Australian Catholic University, Canberra, ACT, Australia; ^3^Eastern Health Clinical School Turning Point, Eastern Health, Richmond, VIC, Australia; ^4^Eastern Health Clinical School, Monash University, Melbourne, VIC, Australia; ^5^School of Medicine, University of Tasmania, Hobart, TAS, Australia; ^6^School of Psychological Sciences, University of Melbourne, Melbourne, VIC, Australia; ^7^San Francisco Veterans Affairs Health Care System (SFVAHCS), San Francisco, CA, United States; ^8^Department of Psychiatry, University of California, San Francisco, San Francisco, CA, United States; ^9^School of Medicine, University of St Andrews, Medical and Biological Science Building, North Haugh, St Andrews, United Kingdom; ^10^Department of Psychology, National University of Singapore, Singapore, Singapore; ^11^Department of Kinesiology and Health, Rutgers University, New Brunswick, NJ, United States; ^12^Department of Medicine, Faculty of Medicine, Imperial College, London, United Kingdom; ^13^Laboratoire de Psychologie Médicale et d’Addictologie, ULB Neuroscience Institute (UNI), CHU Brugmann-Université Libre de Bruxelles (U.L.B.), Brussels, Belgium; ^14^Department of Psychology and Neuroscience, University of North Carolina at Chapel Hill, Chapel Hill, NC, United States; ^15^Department of Psychiatry, Surrey and Borders Partnership NHS Foundation Trust, Leatherhead, United Kingdom; ^16^DVA Medical Center and Department of Radiology and Biomedical Imaging, University of California San Francisco, School of Medicine, San Francisco, CA, United States; ^17^School of Psychology, University of Queensland, Brisbane, QLD, Australia; ^18^Department of Cognitive Psychology, Institute for Cognitive Sciences Studies, Tehran, Iran; ^19^Iranian National Center for Addiction Studies, Tehran University of Medical Sciences, Tehran, Iran; ^20^School of Medicine, Shahid-Sadoughi University of Medical Sciences, Yazd, Iran; ^21^Department of Psychiatry, University of Minnesota, Minneapolis, MN, United States; ^22^Medical School, University of Dundee, Ninewells Hospital, Scotland, United Kingdom; ^23^Department of Psychiatry, Renaissance School of Medicine at Stony Brook University, Stony Brook, NY, United States; ^24^Laureate Institute for Brain Research, University of Tulsa, Tulsa, OK, United States

**Keywords:** neuroscience, addiction medicine, treatment, substance use disorder, fMRI, neuromodulation, neuropsychological assessment, cognitive rehabilitation

## Abstract

Although there is general consensus that altered brain structure and function underpins addictive disorders, clinicians working in addiction treatment rarely incorporate neuroscience-informed approaches into their practice. We recently launched the Neuroscience Interest Group within the International Society of Addiction Medicine (ISAM-NIG) to promote initiatives to bridge this gap. This article summarizes the ISAM-NIG key priorities and strategies to achieve implementation of addiction neuroscience knowledge and tools for the assessment and treatment of substance use disorders. We cover two assessment areas: cognitive assessment and neuroimaging, and two interventional areas: cognitive training/remediation and neuromodulation, where we identify key challenges and proposed solutions. We reason that incorporating cognitive assessment into clinical settings requires the identification of constructs that predict meaningful clinical outcomes. Other requirements are the development of measures that are easily-administered, reliable, and ecologically-valid. Translation of neuroimaging techniques requires the development of diagnostic and prognostic biomarkers and testing the cost-effectiveness of these biomarkers in individualized prediction algorithms for relapse prevention and treatment selection. Integration of cognitive assessments with neuroimaging can provide multilevel targets including neural, cognitive, and behavioral outcomes for neuroscience-informed interventions. Application of neuroscience-informed interventions including cognitive training/remediation and neuromodulation requires clear pathways to design treatments based on multilevel targets, additional evidence from randomized trials and subsequent clinical implementation, including evaluation of cost-effectiveness. We propose to address these challenges by promoting international collaboration between researchers and clinicians, developing harmonized protocols and data management systems, and prioritizing multi-site research that focuses on improving clinical outcomes.

## Introduction

The past two decades have seen significant advances in our understanding of the neuroscience of addiction and its implications for practice [reviewed in (1–3)]. However, despite such insights, there is a substantial lag in translating these findings into everyday practice, with few clinicians incorporating neuroscience-informed interventions in their routine practice (4). We recently launched the Neuroscience Interest Group within the International Society of Addiction Medicine (ISAM-NIG) to promote initiatives to bridge this gap between knowledge and practice. This article introduces the ISAM-NIG key priorities and strategies to achieve implementation of addiction neuroscience knowledge and tools in the assessment and treatment of substance use disorders (SUD). We cover four broad areas: (1) cognitive assessment, (2) neuroimaging, (3) cognitive training and remediation, and (4) neuromodulation. Cognitive assessment and neuroimaging provide multilevel biomarkers (neural circuits, cognitive processes, and behaviors) to be targeted with cognitive and neuromodulation interventions. Cognitive training/remediation and neuromodulation provide neuroscience-informed interventions to ameliorate neural, cognitive, and related behavioral alterations and potentially improve clinical outcomes in people with SUD. In the following sections, we review the current knowledge and challenges in each of these areas and provide ISAM-NIG recommendations to link knowledge and practice. Our goal is for researchers and clinicians to work collaboratively to address these challenges and recommendations. Cutting across the four areas, we focus on cognitive and neural systems that predict meaningful clinical outcomes for people with SUD and opportunities for harmonized assessment and intervention protocols.

## Cognitive Assessment

Neuropsychological studies consistently demonstrate that many people with SUD exhibit mild to moderately severe cognitive deficits in processing speed, selective, and sustained attention, episodic memory, executive functions (EF: working memory, response inhibition, shifting and higher-order functions such as reasoning, problem-solving, and planning), decision-making and social cognition (5–10). Furthermore, neurobiologically-informed theories and expert consensus have identified additional cognitive changes not typically assessed by traditional neuropsychological measures, namely, negative affectivity and reward-related processes (e.g., reward expectancy, valuation and learning, and habits-compulsivity) (11–13).

Cognitive deficits in SUD have moderate longevity, and although there is abstinence-related recovery (14–16), these deficits may significantly complicate treatment efforts during the first 3 to 6 months after discontinuation of drug use. Thus, one of the most critical implications of cognitive deficits for SUD is their potential negative impact on treatment retention and adherence, in addition to clinical outcomes such as craving, relapse, and quality of life. A systematic review of prospective cognitive studies measuring treatment retention and relapse across different SUD suggested that measures of processing speed and accuracy during attention and reasoning tasks (MicroCog test battery) were the only consistent predictors of treatment retention, whereas tests of decision-making (Iowa and Cambridge Gambling Tasks) were the only consistent predictors of relapse (1). A later review that focused on substance-specific cognitive predictors of relapse found that long-term episodic memory and higher-order EF (including problem-solving, planning, and decision-making) predicted alcohol relapse, whereas attention and higher-order EF predicted stimulant relapse, while only higher-order EF predicted opioid relapse (8). Working memory and response inhibition have also been associated with increased risk of relapse among cannabis and stimulant users (8, 17, 18). Additionally, variation in response inhibition has been shown to predict poorer recovery of quality of life during SUD treatment (19). Therefore, consistent evidence suggests that processing speed, attention, and reasoning are critical targets for current SUD treatments, whereas higher-order EF and decision-making are critical for maintaining abstinence. Response inhibition deficits seem to be specifically associated with relapse prediction in cannabis and stimulant users and also predict quality of life (a key non-drug-related clinical outcome) (20).

### Practical Considerations: Characteristics and Needs of the SUD Treatment Workforce

The workforce in the SUD specialist treatment sector is diverse, encompassing medical specialists, allied health professionals, generalist health workers, and peer and volunteer workers (21). For instance, in the Australian context, multiple workforce surveys over the past decade suggest that around half the workforce have attained a tertiary level Bachelor (undergraduate) degree or greater (21–24). Similarly, US and European data has shown that education qualifications in the SUD workforce are lower than in other health services (25). Because the administration and interpretation of many cognitive tests are restricted to individuals with specialist qualifications, this limits their adoption in the sector. In addition, when screening does occur in SUD treatment settings, its primary function is to identify individuals requiring referral to specialist service providers (i.e., neuropsychology, neurology, etc.) for more comprehensive assessment and intervention, rather than to inform individual treatment plans.

Two fields in particular have driven progress in cognitive assessment practice for generalist workers: dementia, with an increasing emphasis on screening in primary care (26, 27), and schizophrenia, where cognitive impairment is an established predictor of functional outcome (28) necessitating the development of a standardized assessment battery specifically for this disorder. In the selection of domain-specific tests for the Measurement and Treatment Research to Improve Cognition in Schizophrenia (MATRICS) standard battery, a particular emphasis was placed on test practicality and tolerability, as well as psychometric quality. Pragmatic issues of administration time, scoring time and complexity, and test difficulty and unpleasantness (such as item repetition) for the client should be considered (28). These domains and issues are particularly relevant for the SUD workforce as well. The dementia screening literature has also emphasized these pragmatic issues, leading to a greater awareness and access to general cognitive screening tools.

### Routine Cognitive Assessments in Clinical Practice

To date, the majority of the published literature on routine cognitive screening in SUD contexts has focused on three tests commonly used in dementia screening (29–34): the Mini-Mental State Examination (MMSE) (35), Addenbrooke’s Cognitive Examination (ACE) (36), and the Montreal Cognitive Assessment (MoCA) (37). Due to their development for application in dementia contexts, these screening tools placed a heavy emphasis on memory, attention, language and visuospatial functioning (34). Multiple studies have demonstrated superior sensitivity of the MoCA and the ACE scales compared to the MMSE (34, 38). It is possible that this arises from the MoCA and ACE including at least some items assessing EF (letter fluency and trails) which are absent in the MMSE. Indeed, this may demonstrate an important limitation of adopting existing screening tools designed for dementia in the context of SUD treatment. It can be argued that cognitive screening is most beneficial in SUD contexts when focused on SUD-relevant domains, rather than the identification of general cognitive deficits. Therefore, current neuroscience-based frameworks emphasise the importance of assessing EF, incentive salience, and decision-making in SUD (13, 28, 39, 40). As such, there is much to be gained by applying a process similar to the MATRICS effort (28, 39, 40) in the SUD field to identify a ‘gold-standard’ set of practical and sensitive cognitive tests that can be routinely used in clinical practice.

### Cognitive Assessment Approaches in SUD Research

The most commonly used cognitive assessment approach in SUD research has been the "flexible test battery". This approach combines different types of tests to measure selected cognitive domains (e.g., attention, EF). Attention, memory, EF, and decision-making are the most commonly assessed domains, although there is a considerable discrepancy in the tests selected to assess these constructs (41). Even within specific tests, different studies have used several different versions; for example, at least four different versions of the Stroop test have been employed in the SUD literature (1). Another commonly used approach is the "fixed test battery", which involves a comprehensive suite of tests that have been jointly standardized and provide a general profile of cognitive impairment. The Cambridge Automated Neuropsychological Test Battery (CANTAB) (42), the Repeatable Battery for the Assessment of Neuropsychological Status (RBANS) (43), the Neuropsychological Assessment Battery (NAB) – Screening Module (44) and the MicroCog^™^ (45) are examples of fixed test batteries utilized in SUD research (30, 46–48), although these too have limited assessment of EF. Another limitation of these assessment modules is the lack of construct validity, as they were not originally designed to measure SUD-related cognitive deficits. As a result, they overemphasize assessment of cognitive domains that are relatively irrelevant in the context of SUD and neglect other domains that are pivotal (e.g., decision-making). A common limitation of flexible and fixed batteries is their reliance on face-to-face testing, normally involving a researcher or clinician, and their duration, which is typically around 60-90 min.

To address this gap, a number of semi-automated tests of cognitive performance have been developed, including the Automated Neuropsychological Assessment Metrics (ANAM, developed by the U.S. Department of Defence), Immediate Post-concussion Assessment and Cognitive Testing battery (ImPACT), and CogState brief battery, have been used more widely, although validation studies to date suggest they may not yet have sufficient psychometric evidence to support clinical use (49–53). Research specifically in addictions has begun to develop and validate cognitive tests that can be delivered in client/participants’ homes or *via* smartphone devices (54) (*scienceofbehaviorchange.org*, 2019). Evaluations of the reliability, validity, and feasibility of mobile cognitive assessment in individuals with SUD have been scarce, but promising (55–57).

Cognitive assessment *via* smartphone applications and web-based computing is a rapidly developing field, following many of the procedures and traditions of Ecological Momentary Assessment (EMA) (56). The flexibility and rapidity of assessment offered by mobile applications makes it particularly suited to questions assessing change in cognitive performance over various time scales (within hours to over months). For example, cognitive performance can be assessed in event-based (i.e., participants-initiated assessment entries), time-based and randomly prompted procedures that were not previously feasible, and or valid, in laboratory testing. While the benefits of mobile testing to longitudinal research, particularly large-scale clinical trials, appear obvious (57), the rapidity and frequency of deployment also provide opportunities to test questions of much shorter delays between drug use behavior and cognition. For example, recent studies have examined if daily within-individual variability in cognitive performance, principally response inhibition, was associated with variable likelihood for binge alcohol consumption (58). Similarly, influencing the immediate dynamic relationship between cognition and drug use has also been used for intervention purposes. Web and smartphone platforms have been used to administer cognitive-task based interventions, such as cognitive bias modification (CBM) training (59–61), where cognitive performance is routinely measured as a central element of interventions that span several weeks. The outcomes of these trials show that mobile cognitive-task based interventions are feasible but not efficacious as in a stand-alone context (58, 61). However, the combination of cognitive bias modification (approach bias re-training) and normative feedback significantly reduces weekly alcohol consumption in excessive drinkers (59).

### Summary of Evidence and Future Directions

A substantial proportion of people with SUD have cognitive deficits. Alcohol, stimulants and opioid users have overlapping deficits in EF and decision-making. Alcohol users have additional deficits in learning and memory and psychomotor speed. Heavy cannabis users have specific deficits in episodic memory and attention. Cognitive assessments of speed/attention, EF and decision-making are meaningfully associated with addiction treatment outcomes such as treatment retention, relapse and quality of life (1). In addition, there is growing evidence that motivational and affective domains are also implicated in SUD pathophysiology and clinical symptoms (8). For example, both reward expectancy and valuation and negative affect have been proposed to explain SUD chronicity (13). However, to date, there have been no studies linking these "novel domains" with clinical outcomes. Thus, it is important to explore the predictive validity of non-traditional cognitive-motivational and cognitive-affective domains in relation to treatment response. While flexible and fixed test batteries are the most common assessment approaches, data comparability is alarmingly low and future studies should aim to apply harmonized methods (41). Remote monitoring and mobile cognitive assessment remain in a nascent stage for SUD research and clinical care. It is too early to make accurate cost-benefit assessments of different mobile methodologies. Yet, their potential to provide more cost-effective assessment with larger and more representative samples and in greater proximity to drug use behavior justifies continued investment into their development.

### Challenges for Implementation Into Practice

One of the main challenges for the cognitive assessment of people with SUD is the disparity of tests applied across sites and studies, and the lack of a common ontology and harmonized assessment approach (13, 62). Furthermore, harmonization efforts must accommodate clinicians’ needs, including brevity, simplicity, and automated scoring and interpretation (10). Mobile cognitive testing is a highly promising approach, although its reliability and validity are influenced by a number of key factors. Test compliance, or lack thereof, seems to be problematic. A recent meta-analysis suggested that the compliance rate for EMA (the standard paradigm to administer mobile cognitive testing) with SUD samples was below the recommended rate of 80% (63). Designs including participant-initiated event-based assessments were associated with test compliance issues, whereas duration and frequency of assessment were not. While the latter finding suggests that extensive cognitive assessment may be feasible with mobile methods, caution is advised with regard to the scope and depth of the data that can be obtained with these brief assessments and the validity of data sets collected (64). Remote methods for assessing confounds such as task distraction, malingering, and "cheating" are not well established or validated. As the capability of smartphones, for example, increases, so will the potential to minimize or control for such variables. Face-recognition and fingerprint technology has been proposed for ensuring identity compliance, although this presents ethical issues regarding confidential and de-identified data collection from samples that engage in illicit drug use (65).

### ISAM-NIG Recommendations for Cognitive Assessment

As the authors of this ISAM-NIG roadmap, we give the following recommendations for future work:

1. *Selecting theoretically and clinically relevant constructs*: We recommend prioritizing constructs that are theoretically implicated in current neurobiological models of SUD [reviewed in (66)] and meaningfully related to SUD treatment response and clinical outcomes [e.g., (1, 67, 68)]. These include attention/processing speed, response inhibition, and higher-order EF/decision-making. Episodic and working memory assessments can be particularly indicated in the case of alcohol and cannabis users (8).
2. *Selecting measures with well-established clinical validity in the SUD population*: We recommend using measures with demonstrated predictive and ecological validity (i.e., their scores predict individual variation in meaningful clinical outcomes such as treatment response, craving, drug use/relapse, and quality of life), in addition to reliability. Unfortunately, few such measures are currently available. The MicroCog test battery and Continuous Performance Test (sustained attention/response inhibition) are highly reliable and excellent predictors of treatment response (1). Delay discounting paradigms and gambling tasks have excellent predictive and ecological validity, but the latter have been criticized for low reliability and construct validity (69). Because the ultimate goal is to incorporate cognitive assessment into clinical practice, we recommend conducting a Delphi consensus study including both cognitive assessment researchers and SUD clinicians to identify a minimum battery of measures with adequate psychometric properties AND clinical significance.
3. *Adopting harmonized cognitive assessment protocols*: We recommend continuing work towards developing a harmonized Cognitive Assessment of Addiction (CAA) battery. This battery should be (1) theoretically grounded in current addiction neuroscience frameworks; (2) brief and easy to administer, to meet the needs and qualifications of the SUD workforce; (3) portable and repeatable, capitalizing when possible on emerging remote monitoring techniques; (4) clinically meaningful in individual-level predictive models, i.e., able to identify risk of cognition-related premature treatment cessation or relapse, cognitive phenotypes relevant for predicting response to different treatment approaches, or changes in cognitive status relevant to treatment progression. The CAA should also address challenges specific to international research collaboration, including culturally-sensitive contents and appropriate translation of instructions.

## Neuroimaging

The development of functional imaging techniques such as Positron Emission Tomography (PET) and functional Magnetic Resonance Imaging (fMRI), has allowed the high-resolution mapping of the brain in-vivo, in people with SUD. This body of work has provided increasing evidence that SUD is associated with alterations in the anatomy and the functional brain pathways ascribed to reward, learning, and EF. Importantly, emerging evidence suggests that neuroimaging versus subjective measures in SUD may predict with greater precision addiction-relevant cognitive processes (e.g., attentional biases) and treatment outcomes (e.g., abstinence) (70–72).

### Neuroimaging Methods and Techniques Applied to SUD

Functional imaging techniques allowed exploration of whether brain dysfunction is implicated in SUD in humans. These create images of brain function by relying on proxies, including metabolic properties of the brain (e.g., oxygen in PET and fMRI, glucose levels in PET) (73). The application of functional imaging has been crucial to reveal the impact of SUD on human brain function in areas ascribed to cognitive processes (e.g., EF, decision-making) and positive and negative emotions (see "Cognitive assessment approaches in SUD research" in the COGNITIVE ASSESSMENT section).

PET studies have also provided early evidence on the neurobiology of SUD (74–77). PET imaging relies on the movement of injected radioactive material to identify whether the metabolic activity of brain regions is related to cognitive functions (73). PET’s invasiveness and high financial costs have resulted in a limited number of studies using it, and its low temporal and spatial resolutions (i.e., 20–40 min required for image generation, with a spatial resolution up to 5 mm^3^) prevented the identification of subtle brain activity alterations in SUD samples (73).

The development of fMRI provided a way to overcome these limitations. Unlike PET, fMRI is non-invasive, promoting feasibility in unpacking the neural correlates of SUD (73). Specifically, fMRI generates information about brain activity by exploiting the magnetic properties of oxygenated and deoxygenated blood (73). Further, fMRI provides information on the brain’s functional activity with higher temporal and spatial resolutions than those of PET, i.e., within seconds and millimeters, respectively (73). These methodological advantages have allowed many studies to map the neural pathways implicated in SUD, while providing information on brain function within a high spatial and temporal resolution. However, a well-described limitation of fMRI analyses is the difficulty to control for multiple tests (i.e., statistical thresholds) and related false positive errors (78). The neuroimaging community has started to implement several strategies to address this limitation (79), but the use of liberal thresholds has probably inflated false positive rates in earlier studies.

Using multi-modal imaging techniques is warranted to further unpack the neural mechanisms of SUD and abstinence. For instance, integrating structural MRI (sMRI) data with Magnetic Resonance Spectroscopy Imaging, an MRI imaging technique that allows investigation of metabolites in the brain, may provide insight into the biochemical changes associated with volumetric alterations in SUD. Further, conducting brief, repeated task-free fMRI studies during treatment/abstinence could provide a better understanding of the impact of clinical changes on intrinsic brain architecture. An advantage of resting-state functional imaging data is the possibility of investigating patterns of brain function without restrictive "forces" on brain function placed by a specific task. Finally, studying SUD with modalities such as Diffusion Tensor Imaging (DTI) may reveal alteration in white matter pathways that connect brain regions that are volumetrically altered. This approach may inform the pathophysiology of volumetric alterations in SUD-relevant brain circuits.

### Brain Systems Implicated in Addiction: Insights From Theory

**Table 1** overviews key neurobehavioral pathways implicated by prominent neuroscientific theories of addiction and a growing body of work. These include neurobehavioral systems implicated in positive valence, negative valence, interoception, and EF (80–86). Abstinence may recover and mitigate such brain alterations and related cognitive functions, e.g., increase in response inhibition capacity, lower stress and drug reactivity, learning new responses to drugs and related stimuli. This notion is yet to be tested using robust neuroimaging methods that, in conjunction with treatment-relevant clinical and cognitive measures, measure and track the integrity of specific neural pathways during abstinence (see examples in **Table 1**).

**Table 1:** Overview of addiction-related neurocognitive constructs and related brain circuits, tasks, and interventions.

	Positive affect, Response (13), (80), (82), (84)	Positive affect, Anticipation (13), (83), (84)	Negative affect (13), (80), (82),	Learning/habit (13), (83), (84)	Cognitive control (13), (82), (83), (84)	Interoception (83), (86)
**Brain circuit**	Medial OFC, ventral striatum	Medial OFC, sgACC (subgenual)	Amygdala	Lateral OFC, Dorsal striatum (Caudate, putamen), Hippocampus	DLPFC, dACC (dorsal), IFG	Insula, posterior cingulate
**fMRI tasks**	Monetary incentive delay (reward receipt) (87), probabilistic reward task (88), activity incentive Delay task (98)	Monetary Incentive delay (reward anticipation) (87), cue-reactivity (90), attentional bias (89)	Cue reactivity (90) during withdrawal, negative or stress cue reactivity	Instrumental reward-gain and loss-avoidance task (89)	Stop Signal (91), Go-no go (92), Stroop (93), PASAT-M (97)	heartbeat counting task (94), visceral interoceptive attention task (95)
**Cognitive**	Reward receipt, response to reward, reward satiation	Motivation, saliency valuation, reward anticipation, drive expectancy, approach/attentional bias	Acute/sustained threat	Stimulus-response conditioned habits, compulsivity, learning reward/loss contingencies	Loss of cognitive control, disinhibition, performance monitoring, action/response selection, low distress tolerance	"Momentary mapping of the body’s internal landscape" (96) during craving and withdrawal
**Behavior**	Experience of reward with drug use, response to substance-free reward	Increased: attention/salience of drugs and related stimuli, reward when anticipating drug use.	Experience of withdrawal, stress, anxiety, anhedonia	Drug use as: repetitive, compulsive drive, conditioned response to seek positive affect & avoid/mitigate negative affect, learnt association with people, situations, places	Drug use even when known as harmful and in response to affective distress	Heightened/lowered awareness to drug-related physical & psychological states; increase distance between cue and behavioral response.
**Intervention strategies**	Decrease reward value of drug (e.g., methadone or nicotine patches), suppression of mPFC with low frequency rTMS or cTBS; increase reward value of drug-free activities (e.g., behavioral activation, physical activity)	Cognitive bias modification, reappraisal training for drug cues, exposure therapy, motivational interviewing, contingency management	Strategies to address negative affect (e.g., behavioral activation and cognitive reappraisal training), medication that counter stress response, rtfMRI neurofeedback on Insula or sgACC	Strategies that weaken conditioned drug behaviors, memory reconsolidation	Strengthen inhibitory/executive control, inhibitory control training (e.g., Go-No-Go), working memory training, goal management training, stimulating DLPFC with anodal tDCS or high frequency rTMS	Mindfulness-based therapies, physical exercise

Columns reflect key neurocognitive constructs for addiction research. Identified constructs also map onto the three domains of the Addiction Neuroclinical Assessment (ANA) (11) framework: Positive affect (response and anticipation), Negative affect, and Cognitive control map directly onto the three domains of ANA (i.e., Incentive salience, Negative affectivity and Executive function). Learning/habit is part of Incentive salience (reward learning); Interoception is at the interface of the three ANA domains. Rows reflect functional neuroimaging methods (e.g., fMRI tasks), cognitive/behavioral assessments, and examples of neuroscience informed intervention strategies aligned with each of the identified constructs.

ACC, anterior cingulate cortex; cTBS, continuous theta burst stimulation; DLPFC, dorsolateral PFC; IFG, Inferior Frontal Gyrus; mPFC, medial PFC; OFC, orbitofrontal cortex; PFC, prefrontal cortex; rtfMRI, real-time functional MRI; rTMS, repeated transcranial magnetic stimulation; tDCS, transcranial direct current stimulation.

The neurobiology of abstinence has been posited to entail two core processes (99). The first is the *restored* integrity of brain function, as drug levels in the central nervous system and bloodstream clear out with abstinence. The second is the *retraining* of neural pathways implicated in cognitive changes that enable abstinence. These include awareness/monitoring of internal psychological/physiological states (e.g., insula), withdrawal and craving (e.g., amygdala); EF (e.g., dorsal prefrontal regions); monitoring conflict between short-term goals (e.g., pleasure from using drugs, ventral striatum) *versus* long-term goals (e.g., abstinence and improved quality of life; anterior cingulate cortex); motivation to use drugs (e.g., orbitofrontal cortex); and learning new responses to drug-related and other stimuli (e.g., lateral prefrontal and dorsal striatal regions) (99).

### Summary of Neuroimaging Evidence in SUD

Most neuroimaging studies to date have mapped dysfunctional neural pathways in SUD. There is a significant lack of work that tracks abstinence-related brain changes over time. This evidence gap prevents neuroimaging studies from informing the identification of treatment targets and clinical practice. It is unclear if abstinence (i) leads to recovery of SUD-related brain dysfunction (i.e., return to pre-drug onset level, or comparable levels to non-drug using controls), (ii) engages additional pathways implicated in abstinence-related cognitive, clinical, and behavioral changes, and (iii) is predicted by specific brain measures assessed pre-treatment. Emerging (but mixed) evidence from standard behavioral (e.g., CBT, Motivational Interview, Contingency Management) and pharmacological treatments that directly affect the central nervous system provides preliminary support for these notions, as reviewed in detail in previous work [see (100–102)]. This section provides an overview of early neuroimaging evidence for brain changes related to abstinence and novel interventions (i.e., cognitive training approaches and mindfulness-based therapies).

#### Neuroimaging Evidence in Abstinence

Abstinence may "reverse" brain dysfunction and volume loss associated with SUD. Studies have observed increased or normalized volumes in global and prefrontal brain regions related to abstinence in people with alcohol use disorder (103) and cocaine and opiate use disorders (104). PET and DTI studies of alcohol and cocaine users showed recovery of brain dysfunction and white matter integrity following heterogeneous abstinence durations, e.g., from about a month (105, 106), to several months (107, 108) and several years (109, 110). Results from fMRI tasks of response inhibition in abstinent users also showed that reduced brain function typically associated with drug use, was "restored" and increased in prefrontal and cerebellar pathways in former *versus* current cigarette smokers (> 12 month abstinent) (111, 112), and in former cannabis users (> 28 day abstinent) *versus* non-users (113).

Emerging (but mixed) evidence showed that abstinence duration was associated with improved integrity (functional and structure) of cortical and prefrontal pathways (109, 111, 114). Additionally, abstinence related neuroadaptations have been associated with substance use levels [e.g., cocaine dose (115)], and performance was improved during cognitive tasks relevant to addiction [e.g., processing speed, memory, EF-shifting (104, 115)]. Thus, abstinence-related brain changes may in part drive treatment relevant outcomes.

#### Neuroimaging Predictors of Abstinence

Several neuroimaging studies have examined whether (structural and functional) brain integrity in SUD predicts abstinence, with promising results. Studies of *brain structure* in people with nicotine and alcohol use disorders reported that increased volume and white matter integrity in prefrontal regions, followed by parietal and subcortical areas, most consistently segregated abstainers *versus* relapsers (116–119). Studies have examined *brain function* using fMRI tasks that engage cognitive domains relevant to treatment response (cue reactivity, attentional bias, error-related activity, reward, and emotion processing) (71, 72, 111, 116, 117, 120–124). These studies provided evidence that the function of fronto-striatal regions in particular, followed by other regions (e.g., cingulate, temporal, insular cortices) discriminated responders *versus* non-responders, relapsers *versus* non-relapsers in cigarette smokers and people with methamphetamine, cocaine and alcohol use disorders (71, 72, 111, 116, 117, 120, 121, 123, 124). Also, the activity of fronto-striatal pathways have been shown to predict alcohol dosage at 6 month follow-up (122). Studies that used other functional imaging techniques such as spectroscopy and PET imaging consistently reported that frontal blood flow and metabolites (i.e., in prefrontal, insular, and cerebellar areas) and the density of dopamine receptors (i.e., in the dorsal striatum) predicted treatment outcome in alcohol users (125, 126) and relapse in methamphetamine users (127).

#### Impact of Cognitive Training Strategies

Novel training strategies that target core cognitive dysfunctions in SUD have shown promise to restore cognitive alterations and help maintain abstinence (128). One example includes cognitive bias modification strategies that reduce attentional biases towards substance related cues [see study in tobacco smokers (129)]. Such strategies may target top-down and bottom-up brain pathways (130) implicated in addiction (131). These include increasing the activity of top-down EF regions that enhance inhibitory control and behavioral monitoring (e.g., dorsal anterior cingulate, lateral orbitofrontal cortex), and decreasing reactivity of bottom-up pathways implicated in reactivity to drug stimuli, and craving (e.g., amygdala).

Early neuroimaging evidence has examined the neuroadaptations that occur pre-to-post-cognitive bias modification training. These findings are revised and discussed in the COGNITIVE TRAINING AND REMEDIATION section below. There is a paucity of neuroimaging research on other cognitive training and remediation approaches, despite promising evidence of neuroplasticity-related changes after cognitive remediation in brain injury (132).

#### Impact of Mindfulness-Based Interventions

Mindfulness-based interventions are being increasingly used for the treatment of SUD (133). Although mindfulness does not use standard cognitive training/remediation approaches, it has shown to improve SUD-relevant cognitive processes such as attention and EF (134) as well as substance use outcomes (i.e., reduced craving, withdrawal) (135). Mindfulness-based interventions engage two key cognitive processes (i) *focused attention*, which consists of paying attention to a specific stimulus while letting go of distractions (e.g., focus on breathing, while experiencing craving) and (ii) *open monitoring*, which refers to the being aware of internal and external stimuli (e.g., acknowledging the experience of stress, craving, and withdrawal, or environmental triggers) with a non-judgmental attitude and acceptance.

The effectiveness of mindfulness-based interventions has been ascribed to improved function of prefrontal, parietal, and insula regions that are implicated in EF and autonomic regulation (133, 136), and down-regulation of reactivity in striatal/amygdala regions implicated in reward, stress, and habitual substance use (136). Only a handful of neuroimaging studies have examined brain changes that occur with mindfulness-based interventions in SUD. This includes a fMRI study in tobacco smokers that showed a 10-session mindfulness-oriented recovery enhancement (MORE) *versus* placebo intervention, decreased activity of the ventral striatum, and medial prefrontal regions during a craving task and an emotion regulation task (137). Most evidence on mindfulness and SUD consists of behavioral studies that showed robust effects on cognition, substance use, and craving. Given the widespread use of mindfulness-based interventions in clinical settings, we advocate the conduct of active placebo-controlled neuroimaging studies that map the neurobiology of mindfulness in SUD.

### Challenges for Implementation Into Practice

Overall, there is a paucity of neuroimaging studies of treatment and abstinence in SUD. The study methods are very heterogeneous which precludes their systematic integration. *First*, there was significant heterogeneity in treatments, with distinct durations and hypothesized neurobehavioral and pharmacological mechanisms of action, and distinct treatment responses across different individuals, SUD and related psychiatric comorbidities. *Second*, control groups varied substantially (e.g., placebo, active control treatment, no control group) and brain changes related to abstinence were compared to different types of controls (e.g., pretreatment baseline in the same group, control group of non-substance users, separate SUD group also assessed post-treatment). *Third*, repeated measures study designs had varying data testing points (e.g., before, during and at varying times post-treatment) that precluded the integration of the study findings and mapping treatment-related, trajectories of brain changes with abstinence/recovery. More systematic evidence is needed to provide sufficient power to measure brain pathways relevant to treatment response and to inform clinically-relevant treatment endpoints. In order to address this gap, the ISAM-NIG Neuroimaging stream recommends the conduct of harmonized, multi-site, neuroimaging studies with systematic testing protocols of relevance for clinical practice. It is hoped that the ISAM-NIG Neuroimaging approach will generate results that can be readily integrated and that increase the power to detect abstinence-related neuroadaptations.

On one hand, the integration of neuroimaging testing into clinical practice can be challenging. MRI scanners are extremely expensive to buy, setup, and run safely, and the acquisition of high-quality brain images requires extensive specialized technical expertise. On the other hand, the availability of MRI scans in many hospitals, universities, and medical institutions, may provide ideal settings to integrate neuroimaging and clinical expertise. MRI scans can be feasible in that they are non-invasive, safe, and can be relatively quick (e.g., anatomical and resting-state brain scans can take <10 min, and some fMRI tasks can last between 10 and 15 min). Outstanding challenges to address remain funding sources, the lack of integration in the theoretical frameworks between basic research, clinical science, and clinical practice. Discipline-specific specialized language and practices can also create barriers. We advocate using team science to develop a harmonized interdisciplinary framework, so that all stakeholders, including clinicians, neuropsychologists, social workers and neuroscientists interact to inform commonly-agreed testing batteries and most profitable directions for future work.

The present review has focused on neuroimaging data mainly acquired through fMRI, allowing for visualization of the brain networks involved in certain conditions (e.g., abstinence vs. relapse). However, it should be noted that the coarse temporal resolution of such techniques (1–2 s) impedes determination of the temporal activation sequence (in the order of the ms), allowing the specific brain activation patterns to be correlated with the various cognitive stages involved in the investigated processes [e.g., (138)]. Other tools, such as cognitive event-related potentials (ERPs) in particular, might be more suitable for this purpose (139). Nowadays, different studies reveal that specific ERP components tagging specific cognitive functions (mainly cue reactivity and inhibition) may be used as neurophysiological biomarkers for addiction treatment outcome prediction (140). Such data may be of great value to clinicians for the identification of cognitive processes that should be rehabilitated on a patient-by-patient basis through cognitive training and/or brain stimulation. However, despite technical facilities (cheap tool easily implementable in each clinical care unit), several decades of research, and clinical relevance, ERPs like other neuroimaging modalities have yet to be implemented in the clinical management of SUD.

### ISAM NIG Recommendations for Neuroimaging

We aim to map how advanced multimodal neuroimaging tools—coordinated with relevant clinical and cognitive measures agreed upon with a large multidisciplinary team of experts in the field—can be used to track the neurobiological mechanisms of addiction treatment. As the authors of this ISAM-NIG roadmap, we give the following recommendations for future work:

1. Neuroimaging testing should be *harmonized* with clinical and cognitive tools mapping overlapping systems (see example in **Table 1**).
2. Neuroimaging testing should be *feasible* and rely on short and robust imaging protocols that recruit specific brain pathways implicated in relevant clinical and cognitive features of addiction (e.g., craving, attentional bias, cognitive control).
3. Neuroimaging protocols may also incorporate *neuroimaging measures of brain integrity other than those included in the harmonized protocols when focused on discovery science* (e.g., new fMRI tasks that target novel cognitive constructs, new neuroimaging techniques that test distinct properties of brain integrity). This would mitigate the risks that complete harmonization around existing neuroimaging measures and neurobiological models of addiction would stifle new knowledge. We cannot exclude that current neuroimaging techniques and theories of addiction may not be an accurate/valid representation of brain changes that occur with SUD treatment.
4. Imaging testing batteries should be *amenable to repeated testing* so that changes over time can be tracked (i) *prospectively*, to examine if baseline imaging measures predict follow up outcomes assessed 1+ times at the end of treatment, (ii) *longitudinally*, to track individual trajectories of brain and behavioral change before, during and after treatment, (iii) using rigorous *double-blind randomized controlled studies* to map treatment-specific effects in distinct substance and behavioral addictions.
5. *Multi-site* neuroimaging studies using shared protocols will be necessary to gain sufficient power to track heterogeneity of treatment responses between individuals SUD, to validate the protocols and test their reliability. There are excellent examples of successful international collaborations that are already in place in this area, such as ENIGMA-Addiction (141). We aim to leverage these existing collaboration initiatives to increase neuroimaging methods reliability and validity and studies sample size and representativity, and to expand them by incorporating more clinical researchers and clinicians.
6. As treatments often consist of individual and combined interventions, the distinct and cumulative effects on brain changes should be examined. In addition, investigating moderating roles of age and sex differences on these abstinence-related neuroadaptations is critical. Indeed, younger and older people with SUD may show lower and greater vulnerability to aberrant neurobiology (142). People with different ages and sex may show distinct neuroplastic changes with abstinence and these are largely unknown (99, 143, 144).
7. Brain indices from neuroimaging testing should be *examined in relation to treatment response variables*, whether measured as categories (e.g., responders vs. non-responders, relapsers vs. non-relapsers) or as discrete measures of addiction (severity of addiction symptom scores, number of relapses, duration of abstinence, amount of substance used) and related mental health, cognitive and quality of life outcomes (e.g., stress, mood, socio-occupational functioning).

## Cognitive Training and Remediation

Despite recent advances in psychological and pharmacological interventions for SUD, relapse remains the norm. A recent meta-analysis of 21 treatment outcome studies conducted between 2000–2015 found that fewer than 10% of treatment seekers were in remission (i.e., did not meet SUD diagnostic criteria for the past 6 months) in any given year following SUD treatment (145). The past decade has seen a proliferation of cognitive training (CT) intervention trials aimed at remediating or reversing substance-related cognitive deficits (146). However, their implementation into clinical practice is almost non-existent, despite promising results and now having more flexible, precise, engaging and convenient modes of delivery (i.e., computer, web and mobile application-based approaches). Gathering more data in this still-developing area is essential to facilitate translation. Even the most widely tested training interventions, such as cognitive bias modification, need more data to fully appraise their benefit for addiction treatment (147). This section summarizes recent advances in CT, identifies limitations in the evidence base, and highlights priorities and directions for future research to bridge the gap between science and practice. Current CT approaches can be broadly divided into: general cognitive remediation, working memory training (WMT), inhibitory control (or response inhibition) training (ICT), and cognitive bias modification (CBM).

### Cognitive Remediation

In SUD, general cognitive remediation approaches such as cognitive enhancement therapy (CET) and cognitive remediation therapy (CRT) aim to reduce substance use (148–150) and craving (151) by targeting EF and self-regulation. Cognitive remediation has been shown to improve cognition in domains of working memory (WM), verbal memory, verbal learning, attention, and processing speed (151–154). Positive outcomes have also been shown to be associated with increased neuroplasticity in emotion regulation-related fronto-limbic networks in individuals with schizophrenia and co-morbid SUD (155). A recent study delivered 12 two-hour group sessions of clinician-guided CRT and computerized CT (Lumosity) (156) over 4 weeks to a sample of female residents completing residential rehabilitation and found significant improvements in EF, response inhibition, self-control, and quality of life relative to treatment as usual (TAU) (157). Similar research has reported comparable improvements in cognitive functioning following CRT (150, 151) and CET (148), and improved cognitive functioning has been associated with reduced substance use at 3- and 6-month follow-ups (148, 150). Importantly, CET and CRT also demonstrate preliminary efficacy for SUD patients with cognitive impairments (e.g., schizophrenia, past head injury) (148, 157). However, their duration, intensity, and high cognitive demand—coupled with a current paucity of large-scale, methodologically rigorous clinical trials—may currently preclude their widespread implementation in clinical settings.

Another manualized therapist-assisted group intervention is Goal Management Training (GMT), which trains EF and sustained attention and emphasizes the transfer of these skills to goal-related tasks and projects in everyday life. When combined with mindfulness meditation, GMT has been found to significantly improve WM, response inhibition and decision-making in alcohol and stimulant outpatients relative to TAU (158) and more recently also in polysubstance users in a therapeutic community (159). A meta-analysis of GMT more broadly concluded that it provides small to moderate improvements in EF which are consistently maintained at 1–6 month follow-ups (160). As such, GMT is likely to be an effective candidate cognitive remediation approach for SUD treatment; however, substantially more research is needed to validate this assertion, particularly regarding the translation of cognitive improvements into improved substance use outcomes.

### Working Memory Training (WMT)

The most widely researched EF training intervention, WMT (e.g., Cogmed, PSSCogRehab) (161, 162) requires participants to repeatedly manipulate and recall sequences of shapes and numbers through computerized tasks that become increasingly difficult over time (i.e., they are adaptive to the individual’s performance). WMT aims to extend WM capacity, so individuals can better integrate, manipulate, and prioritize important information, with the aim of supporting more adaptive decision-making that leads to reduced substance use (163). Relative to many other approaches, WMT is intensive, typically requiring 19–25 days of training and as such, retention is often poor (164). While WMT has been shown to lead to improvements in near-transfer effects (i.e., improved performance on similar WM tasks), there is limited evidence supporting far-transfer effects of WMT on other measures of EF and importantly, on substance-related outcomes (165). Reduced alcohol consumption 1 month after training was reported following WMT in heavy drinkers (163), but most studies have failed to demonstrate or even measure changes in substance use (165). For example, non-treatment seekers with alcohol use disorder who were trained with Cogmed showed improved verbal memory but no clinically significant reductions in alcohol consumption or problem severity (166). While a study of treatment-seekers improved WM and capacity to plan for the future (i.e., episodic future thinking) on a delay discounting task, there was no measurement of substance use outcomes (167). Similarly, studies of methadone maintenance (168) and cannabis (169) have found no evidence of far-transfer effects (e.g., delay discounting), although Rass et al. (168) showed WMT-related reductions in street drug use among methadone users. Other forms of WMT (e.g., n-back training) have reported similar near-transfer but not substance-use-related findings with methamphetamine patients (170) and a mixed group of substance use patients (alcohol, cannabis, cocaine) (164). As such, the greatest limitation in the WMT literature is the failure to consistently examine substance use outcomes and therefore there is insufficient evidence at this time to support the utility of WMT as an effective adjunctive treatment for SUD.

### Inhibitory Control Training (ICT)

Since deficits in inhibitory control are associated with increased drug use (171–174), ICT aims to bolster inhibitory control through the repeated practice of tasks [e.g., go/no-go (GNG), stop-signal task]. Such tasks require individuals to repeatedly inhibit prepotent motor responses to salient stimuli (172). In a seminal study, a beer-GNG task which trained heavily drinking students to inhibit responses to "beer" stimuli resulted in significantly reduced weekly alcohol intake relative to students trained towards "beer" stimuli (175). A recent RCT of 120 heavily drinking students found that a single session of either ICT or approach bias modification (ApBM, described below) led to significant reductions in alcohol consumption relative to matched controls (176). Similarly, Kilwein et al. (177) found that a single session of ICT (GNG) reduced alcohol consumption and alcohol approach tendencies in a small sample (n = 23) of heavily drinking men (177). Despite these promising findings, each of the aforementioned ICT studies used community samples, and it has not yet been established whether these results will generalise to treatment seekers.

Two meta-analyses recently concluded that ICT leads to small but robust reductions in alcohol consumption immediately after training (178, 179). Di Lemma and Field (176) reported reduced alcohol consumption in a bogus taste test after a single session of ICT or cue-avoidance training (approach bias modification). Others have observed reduced alcohol consumption 1 and 2 weeks after ICT (163, 177, 180). These findings highlight the promise of ICT though there remains a paucity of research assessing long-term drinking outcomes outside of laboratory settings. Future studies of ICT with clinical populations should consider testing multi-session approaches akin to WMT. To date, few studies have trialled multi-session ICT: One found it to be ineffective (58) for heavily drinking individuals, while another found that 2 weeks of ICT resulted in modest reductions of alcohol consumption among individuals with AUDs, compared to WMT or a control condition (181).

### Cognitive Bias Modification (CBM)

CBM aims to directly interrupt and modify automatic processes in response to appetitive cues. Attentional bias modification (AtBM) aims to modify the preferential allocation of attentional resources to drug cues by repeatedly shifting attention to neutral or positive (non-drug) cues and away from drug-related cues. Despite several null findings (182), significant effects have included the reduction of alcohol consumption in non-treatment seeking heavy or social drinkers (183, 184). Among treatment seekers, five sessions of AtBM have been shown to significantly delay time to relapse (but not relapse rates) relative to controls who received sham training (185). Similarly, six sessions significantly reduced alcohol relapse rates at a one-year follow-up relative to a sham training condition in a sample of treatment seekers with AUD (186). Among methadone maintenance patients, AtBM reduced attentional bias to heroin-related words, temptations to use, and number of lapses relative to TAU (187). However, among individuals with cocaine use disorder, it failed to reduce attentional bias, craving, and cocaine use (188). Likewise, 12 sessions of AtBM vs. sham training during residential treatment for methamphetamine use disorder failed to reduce craving and preferences for methamphetamine images (189). A systematic review of alcohol, nicotine, and opioid AtBM studies concluded that despite numerous negative findings in the literature, eight out of 10 multiple-session studies resulted in reduced addiction symptoms (particularly for alcohol), but without concomitant reductions in attentional bias (190).

Approach bias modification (ApBM), which uses the Approach Avoidance Task, requires an avoidance response to drug cues (pushing a joystick, shrinking image size) and an approach response (pulling a joystick, enlarging image size) to non-drug cues. Several trials have examined alcohol ApBM, with evidence that short-term abstinence is increased by up to 30% with four consecutive training sessions during inpatient withdrawal (32) and by 8%–13% at 12-month follow-up (186, 191, 192). Alcohol ApBM has demonstrated relatively consistent, moderate reductions in drinking behavior when delivered to clinical populations (193), and it was even added to the German guidelines for the treatment of AUD (194).

Early neuroimaging evidence has examined the neuroadaptations that occur pre-to-post-cognitive bias modification training. This work has focused on two samples of abstinent alcoholics undergoing an fMRI cue-reactivity task (alcohol *versus* soft drink stimuli) (61, 195). Participants showed higher baseline reactivity to alcohol cues within the amygdala/nucleus accumbens and the medial prefrontal cortex, respectively (61, 195). The same samples, following a 3-week implicit avoidance task (versus placebo), showed reduced amygdala and medial prefrontal reactivity (61, 195). Notably, these brain changes were associated with reduced craving and approach bias to alcohol stimuli (61, 195) but not abstinence 12 months later. While preliminary, these findings suggest that neuroadaptations associated with cognitive bias modification have clinical relevance and warrant replication in larger SUD samples using robust, active placebo-controlled designs.

To date, only one study has been published that trialled ApBM in an illicit drug-using sample of non-treatment-seeking adults with cannabis use disorder (N = 33). Relative to sham-training, four sessions resulted in blunted cannabis cue-induced craving (196) but not less cannabis use. Overall, evidence suggests that ApBM is associated with reduced approach bias and reduced consumption behaviors for alcohol, smoking, and unhealthy foods (197). Recently, six sessions of ApBM delivered to 1,405 alcohol-dependent patients significantly reduced alcohol relapse rates at a 1-year follow-up relative to a sham-training condition (186). However, as these reductions were also observed following AtBM and a combined AtBM and ApBM condition, the authors concluded that all active CBM training conditions had a small but robust long-term effect on relapse rates.

Finally, a meta-analysis of alcohol and smoking CBM studies (both AtBM and ApBM) showed a small but significant effect on clinical outcomes for alcohol (but not smoking), but a lack of evidence that reduced approach bias led to improved outcomes (198). This assertion was challenged by Wiers et al. (193) who noted that the review conflated proof-of-principle lab-studies and clinical RCTs and different samples (e.g., treatment-seeking alcohol dependent individuals vs non-clinical student populations). Importantly, these populations likely have differences in motivation/awareness for receiving an intervention to reduce alcohol use, which could explain inconsistencies in the reported effectiveness of CBM across populations (193).

### Summary of Evidence and Future Directions

Currently CBM, particularly ApBM, appears one of the most promising approaches for individuals seeking treatment for AUDs; however, its effectiveness for other drugs (aside from tobacco) is yet to be established. The most extensively trialled CT approach is WMT, which has shown promising results in alcohol and stimulants users. However, its high cognitive demand, training intensity, and apparent lack of far-transfer effects limit its application to clinical populations. ICT holds much promise for reducing alcohol consumption in heavy drinkers, but requires testing in treatment-seekers. Finally, more intensive group-based approaches such as CRT/CET and GMT may improve EF and quality of life; however, their impact on substance use outcomes remains largely untested. Synergistic approaches now warrant exploration. Indeed, a study that combined WMT and AtBM (199) has shown promising feasibility and improved EF, though substance use outcomes were not assessed. It may also prove fruitful to adopt staggered CT approaches, capitalizing on the brain^,^s capacity to repair itself (neuroplasticity) during withdrawal, early and later abstinence by strengthening cognitive control (e.g., using ICT) and dampening cue-reactivity (e.g., using CBM), prior to engaging in more intensive and cognitively demanding but ecologically valid group training for more extensive remediation (e.g., using GMT).

### Challenges for Implementation Into Practice

While there may be logistical challenges to the adoption of CT in clinical practice (e.g., cost, lack of time, training requirements, etc.), the main impediment to implementing CT in clinical practice is the absence of robust evidence for treatment success of any one particular approach. This is largely due to the vast heterogeneity of studies, particularly regarding differences in treatment settings, samples (clinical vs. non-clinical populations), cognitive intervention approaches, number and duration of training sessions, targeted mechanisms, targeted drugs of concern and varying primary outcome measures. Similarly, the absence of brief, ecologically valid, easily-administered measures of cognition precludes the identification of candidates who are most likely to benefit from CT (e.g., individuals with the poorest WM or the strongest attentional bias). As such, the evidence base for CT remains hampered by (1) the marked lack of studies on clinical populations, (2) the counter-intuitive neglect of assessing relevant substance use outcomes, (3) the lack of adequately-powered RCTs, (4) the limitations of research designs, (5) lack of attention to individual-level trajectories of cognitive improvements in relation to substance use and quality of life outcomes (precision medicine approach), and (6) a simple focus on direct relations between cognitive deficits and outcomes without considering person and environmental mediators and moderators of this relation (14). Despite positive signals from proof-of-concept studies and pilot RCTs, they require replication and testing with suitable control conditions in order to demonstrate their applicability in clinical settings. These limitations highlight the need for a harmonization approach that promotes greater standardization in cognitive training protocols and assessment of its effectiveness (i.e., routine assessment of substance use outcomes). Since the software and manuals of some of the most promising interventions (e.g., CBM, GMT) are well-developed and reproducible, we should advance towards optimized shared protocols that can promote international collaborations and multi-site studies. These recommendations will elucidate what works, for whom and under what conditions (i.e., identifying neurocognitive phenotypes). This knowledge will then guide the adoption of CT to improve outcomes for people seeking treatment for SUD.

### ISAM-NIG Recommendations for Cognitive Training and Remediation

As the authors of this ISAM-NIG roadmap, we give the following recommendations for future work:

1. *The a priori publishing of research protocols*: To improve the consistency of cognitive training trials we encourage the publishing of research methodologies and protocols. This will permit replication studies to aid the consolidation of a disparate evidence base and help determine the optimal training duration and frequency to be implemented in real world clinical settings.
2. *Adopting consistent training paradigms and tailored, context-relevant stimuli:* A challenge for CBM research is the absence of consensus on optimal sham training conditions (e.g., matched stimuli with different push-pull contingencies) and optimal approach stimuli (e.g., whether to use neutral stimuli or healthier alternatives such as non-alcoholic beverages) (200). In the context of both CBM and ICT, utilizing personalized/tailored stimuli may increase engagement and effectiveness. For avoidance or "no-go" stimuli this might involve only using beverage types/brands that are regularly consumed by an individual, or images of illicit drug use and paraphernalia reflecting their preferred route of administration. Similarly, approach or "go" stimuli could encompass positive motivational images representing an individual’s personal goals, values, and aspirations (family, employment, hobbies, etc.), which are drawn on heavily in most psychosocial interventions. Furthermore, co-design with consumers and end-users is a fundamental step to developing interventions that will be implemented successfully in practice.
3. *Ensuring targeted constructs are measured in cognitive training trials:* Future research protocols must adopt pre- and post-intervention measures that will elucidate changes in targeted mechanisms, thereby integrating neuroscience into addiction treatment. Importantly, these protocols should enable moderation and mediation analyses using psychophysiological measures (e.g., EEG, skin-conductance) in order to address issues regarding the notorious lack of reliability of traditional measures (e.g., the implicit association task and the approach avoidance task) (192, 201, 202) and thereby more accurately identify individuals most likely to benefit from adjunctive approaches.
4. *Adopting and standardizing SUD-related outcome measurement*: Future research needs to test cognitive interventions in real-world clinical settings and assess meaningful SUD clinical outcomes (i.e., reduced substance use, reduced cue-craving). Clear evidence of reduced harm and consumption is likely to appeal to both clinicians and individuals under their care, thus driving this improved addiction treatment effort.

## Neuromodulation

The exponential growth in our understanding of the neural circuits involved in drug addiction over the last 20 years (3, 203–205) has been accompanied by the introduction of non-invasive brain stimulation technologies (NIBS) capable of modulating brain circuits externally (outside of the skull), such as transcranial magnetic stimulation (TMS) and transcranial electrical stimulation (tES). Technical advances in NIBS has increased hopes to find clinical applications for NIBS in addiction medicine (206). New FDA approval of NIBS technologies in depressive and obsessive-compulsive disorders, which have overlapping brain circuits with SUD, has raised these expectations to a higher level. There are other emerging areas of NIBS for addiction medicine, such as focused ultrasound stimulation (FUS) and transcranial nerve stimulation (tNS). Furthermore, other technologies exist that target neural circuits noninvasively that can be classified as "neuromodulation", such as fMRI- or EEG-neurofeedback (NF), whereby individuals can change their own brain activity in real time using a brain-computer interface. However, this section will primarily focus on tES/TMS/NF. We will review potential targets, ideal scenarios, and complexities in the field of neuromodulation for addiction treatment and then conclude with a few recommendations for future research.

### Potential Targets for Neuromodulation

Targets in the field of neuromodulation should be defined across multiple levels, from behavior, cognitive process, and neural circuit. The NIMH research domain criteria (RDoC) have provided a research framework for mental health disorders that include these levels of targets for neuroscience-informed interventions including neuromodulation. While this framework was not specifically designed for addiction science, it is still a helpful resource. In RDoC terminologies, three main domains are more frequently considered for addiction medicine: *positive valence*, *negative valence*, and *cognitive systems* with a predominant focus on EF (13, 207). Within the positive valence domain, non-drug and drug-related reward processing (drug craving) are the most favorable multi-level targets for addiction treatment. Within the negative valence domain, acute or chronic withdrawal/negative reinforcement, anhedonia, and negative mood/anxiety comorbidities should be considered. EF with a broad definition has also potential to be targeted in neuromodulation (208). For more details, please see **Table 1**.

### Brain Stimulation Studies in SUD

There is a trend of reporting positive results in tDCS and rTMS trials in SUD that is being reflected in systematic reviews and meta-analysis. In a meta-analysis published in 2013 on 17 eligible trials, Jansen, et al., reported that rTMS and tDCS on DLPFC could decrease drug craving (209). A meta-analysis of 10 rTMS studies identified a beneficial effect of high-frequency rTMS on craving associated with nicotine use disorder but not alcohol (210). Another meta-analysis published in 2018 by Song, et al., including 48 tDCS and rTMS studies targeting the DLPFC, reported positive overall effects on reducing drug craving and consumption with larger effect for multi-session interventions compared to single-session interventions (211). A recent meta-analysis with 15 studies using tDCS among nicotine dependents reported positive effect on craving and consumption (212). However, there is a large variation in methodological details (mainly ignored in meta-analyses) that makes it hard to find trials replicating previous findings using same stimulation protocols. Some of these methodological variations are being introduced below with few examples.

**Figure 1:**
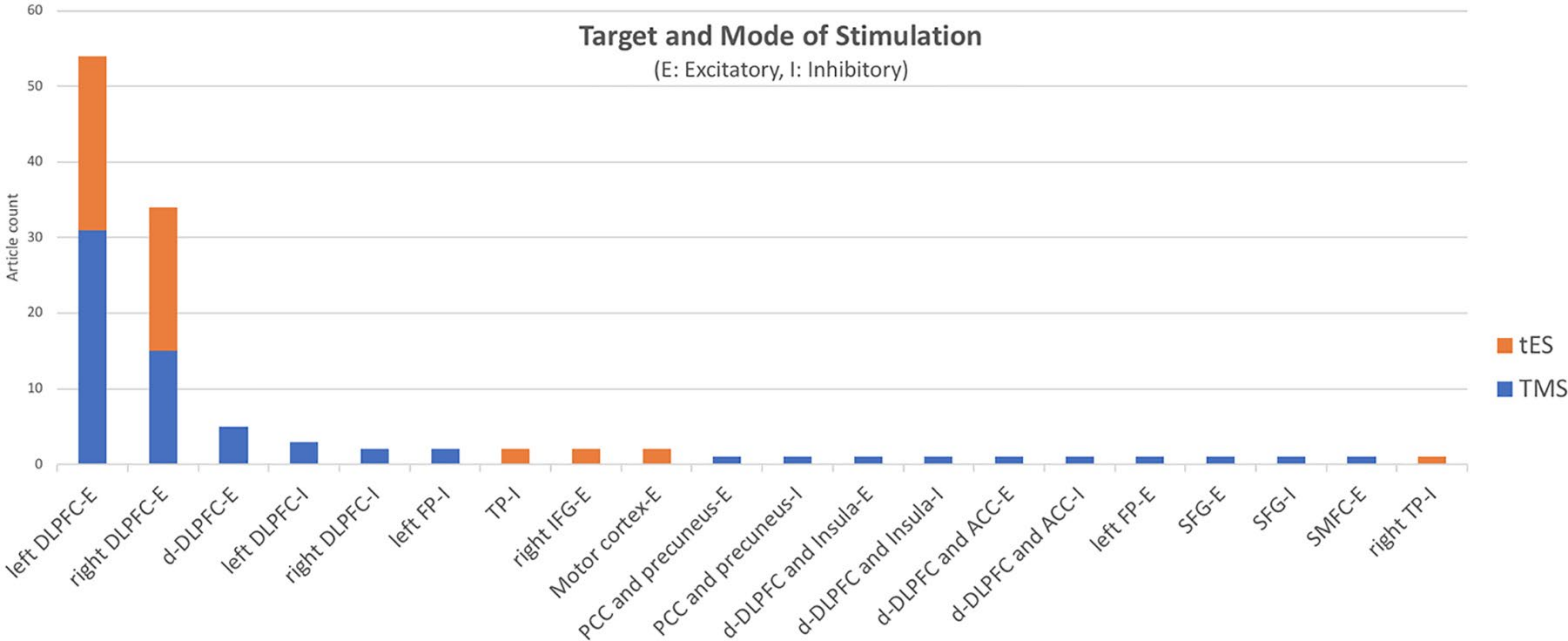
Brain areas targeted with inhibitory (i) and excitatory (e) protocols in 96 tES/TMS studies among people with substance use disorder (as of May 1, 2019) (ACC, anterior cingulate cortex; DLPFC, dorsolateral prefrontal cortex; FP, frontal pole; IFG, inferior frontal gyrus; PCC, posterior cingulate cortex; SFG, superior frontal gyrus; SMFC, superior medial frontal cortex; tES, transcranial electrical stimulation; TMS, transcranial magnetic stimulation; TP, temporoparietal).

**Figure 1** depicts the distribution of published tES/TMS studies based on their target areas. Most but not all published tES/TMS studies (90%) have targeted the DLPFC in order to indirectly target other areas within the EF network or other limbic/paralimbic areas through their connections to the DLPFC. As an example, Terraneo et al. showed that applying 15-Hz stimulation to the left DLPFC can reduce self-reported craving [visual analogue scale (VAS)] and cocaine use (urinalysis) among patients with cocaine use disorder randomized to receive active or sham repetitive TMS (rTMS) (213). In another study, Yang et al. showed that electrical stimulation over the DLPFC helps lower cigarette craving in nicotine-dependent individuals (214). Participant smokers underwent 1 session of real and sham transcranial direct current stimulation (tDCS) in a cross-over setting with 30 min duration and 1-mA intensity. There are studies targeting other areas than the DLPFC within the frontal cortex, such as inferior frontal gyrus, ventromedial prefrontal, or middle frontal cortices. As an example, Kearney-Ramos et al. demonstrated that applying continuous theta burst stimulation (cTBS) as a type of TMS to the ventromedial prefrontal cortex could attenuate the cue-related functional connectivity (215). In another study, Ceccanti et al. found out that deep TMS (dTMS) on the medial prefrontal cortex (MPFC) decreased craving and alcohol intake in people with alcohol use disorder. There are also studies targeting motor cortex and temporoparietal areas which have shown that tDCS reduces behavior in tobacco users. To conclude (as shown in **Figure 1**), the distribution of international resources across all these circuit/process/behavior targets provides interesting explorative results to date. Ignoring these methodological variations could result in positive results in meta-analysis reports. However, considering these methodological details would make it hard to introduce a stimulation protocol with enough evidence for clinical use. There is a critical need in the international NIBS research community to focus on one or two main targets to explore any potentially replicable effects that could determine suitable avenues for clinical application.

Application of other areas of NIBS such as FUS, tNS in addiction medicine is limited to a few case reports. Beyond NIBS, invasive brain stimulation technologies like deep brain stimulation (DBS) are only just emerging as approaches in addiction medicine with only a few case reports or pilot trials in the literature. Consequently, the lack of robust evidence for invasive neuromodulation precludes any judgment regarding its clinical utility.

### Challenges for Implementation Into Practice

**Figure 2:**
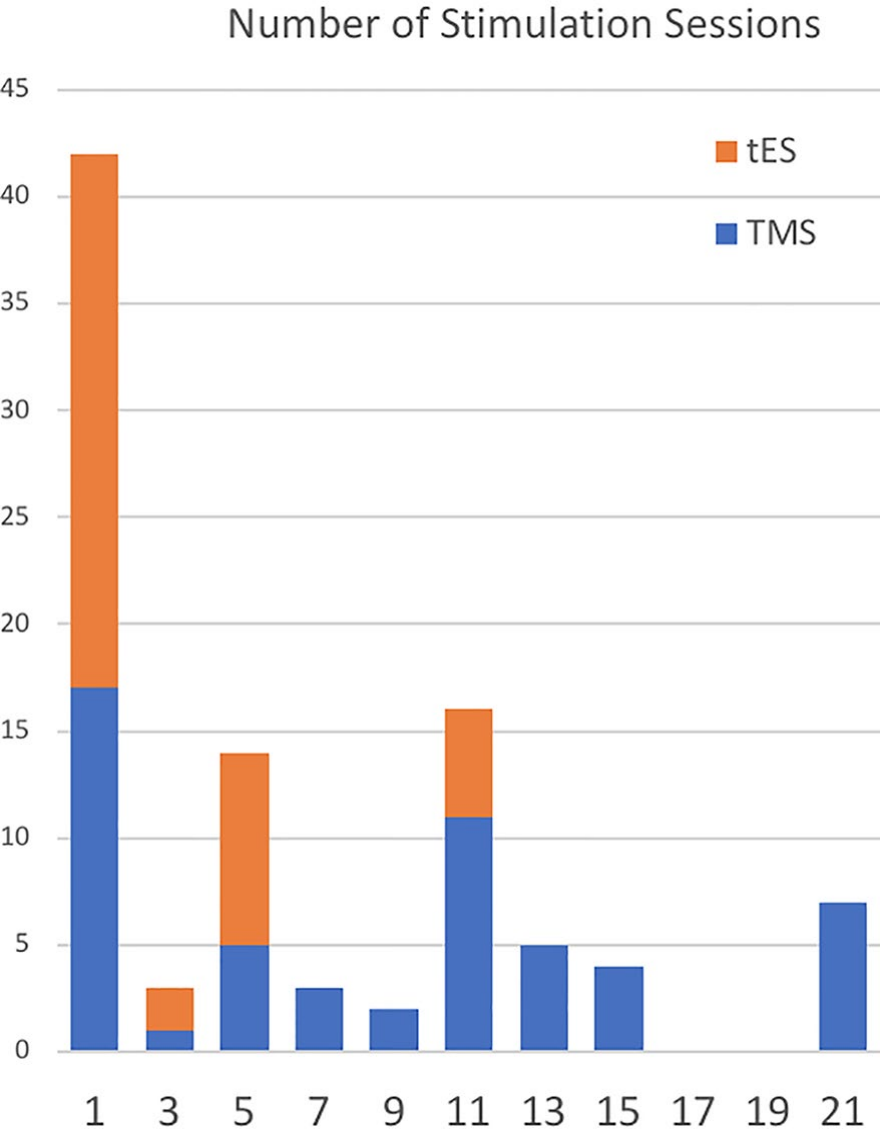
Number of sessions in 96 TMS/tES studies among people with substance use disorder. Around half of the published studies in the field have used just a single session of intervention (as of May 1, 2019). tES, transcranial electrical stimulation; TMS, transcranial magnetic stimulation.

**Figure 3:**
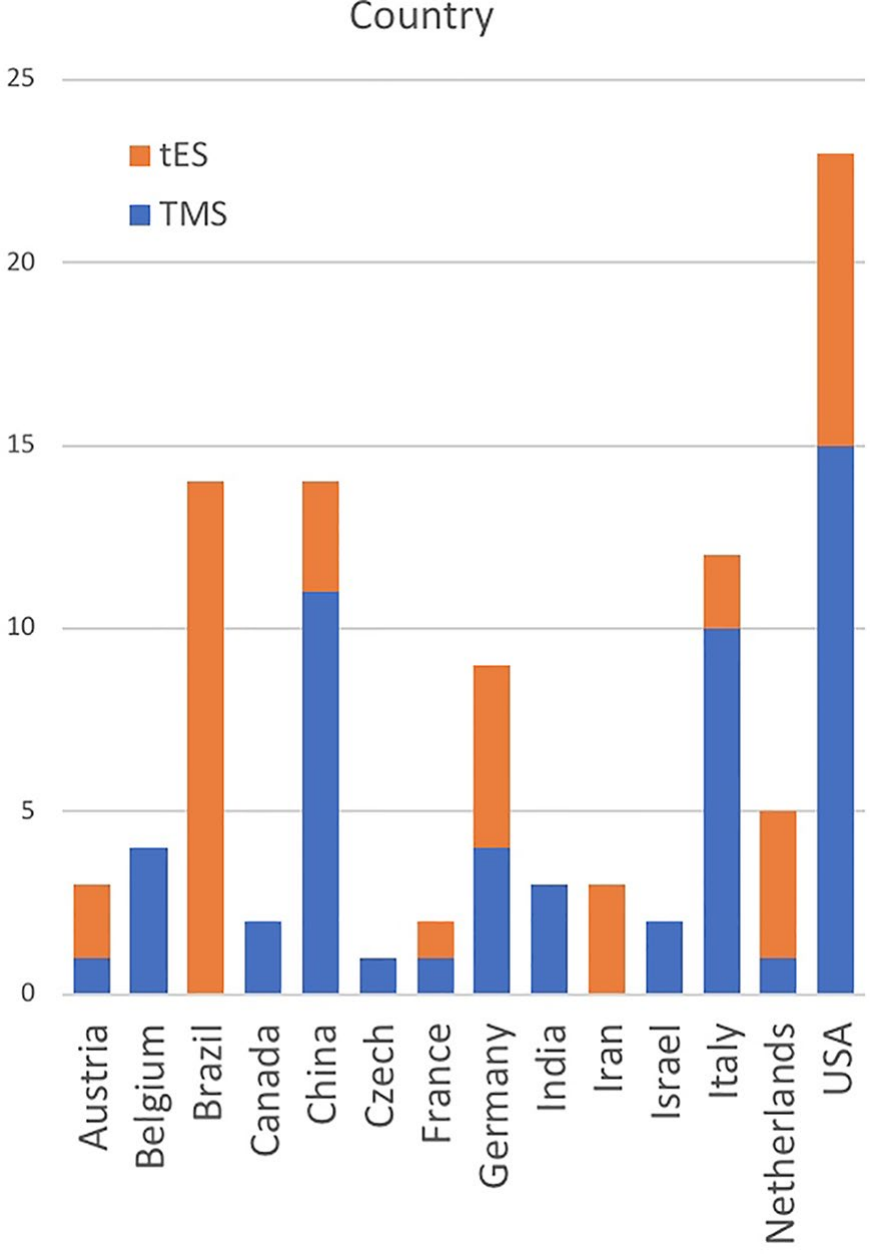
International contribution to the published evidence with tES/TMS in people with substance use disorder. Contribution of 14 different countries (as of May 1, 2019) in the filed confirms the importance of international partnership to improve quality of research in the field. tES, transcranial electrical stimulation; TMS, transcranial magnetic stimulation.

There are 96 original tES/TMS publications in addiction medicine as of May 1, 2019 mainly reporting positive results with one to over 20 sessions of stimulation (**Figure 2**). Large space of methodological parameters to select from, small sample sizes, and lack of replication across different labs make it difficult to draw firm conclusions regarding its effectiveness. Published tES/TMS evidence for addiction treatment has been generated by labs in 14 countries so far (**Figure 3**). To focus these efforts, there is a need for an international roadmap to harmonize the current activities in the field across the world using methodologically rigorous designs. We hope ISAM-NIG along with other international collaborative networks like International Network of tES/TMS Trials for Addiction Medicine (INTAM) can serve to develop and navigate this roadmap. The ISAM-NIG neuromodulation roadmap should also align with ISAM-NIG roadmaps in other areas like brain imaging, cognitive assessments or cognitive training, and this publication is the first attempt at this initiative. These domains of clinical addiction neuroscience can then work hand-in-hand to create tangible outcomes in daily clinical practice. The challenges for implementing neuromodulation studies into practice are summarized below:

1. *How to move beyond single session interventions:* 44% of the tES/TMS studies have recruited a single session of intervention to investigate potential effects to then move forward to multiple session studies (**Figure 2**). By comparison, most of the medications, we use in daily clinical practice in psychiatry today probably do not show significant effects with a single dose. Even adding a sensitive biomarker like a human brain mapping measure using fMRI will not be sufficient for a “no-go” or “fast-fail” decision. In a recent trial with NIMH fast-fail framework, 8 weeks of medication was being considered as the minimum dosage of intervention (216). Meanwhile, running multi-session trials is costly and decisions between the wide range of available parameters to apply and measure are complex.
2. *How to narrow down key brain targets and relevant SUD-relevant cognitive processes/behaviors:* There is a wide range of potential targets for neuromodulation. There is not a consensus on a framework that specifically defines (i) key neuromodulation targets, (ii) their relevant substance use, cognitive, and clinical outcomes, as different brain pathways are ascribed to heterogeneous neurobehavioral processes (**Table 1**), (iii) measurement instruments of desired outcomes with highest psychometric properties.
3. *How to find the best target population/timing for intervention/contextual treatment:* Timing of neuromodulation intervention [before treatment, before initiating abstinence, during early abstinence (detoxification), after early abstinence (maintenance)] and contextual treatment (pharmacotherapies, psychosocial interventions, cue exposure, cognitive remediation, etc.) in parallel to neuromodulation are important areas for future explorations with specific considerations in different SUDs.
4. *How to optimize the large parameter space within each NIBS technology at the individual level:* There is a new effort to optimize the stimulation parameter for each individual subject based on their subjective responses or objective biomarkers in closed-loop stimulation. Bayesian optimization protocols have introduced an interesting area with initial positive response with transcranial alternating current (tACS) stimulation (217). Additionally, personalized brain treatment targets can be identified using neurofeedback machine learning approaches that discriminate distinct patterns of brain function within each individual, instead of *a priori* brain regions (or their connectivity) across various individuals (218).

### Neurofeedback Studies in SUD

Real-time neurofeedback allows online voluntary regulation of brain activity and has shown promise to enhance ascribed cognitive processes in health and psychopathology (219–221). Participants can monitor their brain function in real time through a brain computer interface (BCI), typically showing a thermometer representing the "temperature" of which increases/decreases in real time, to reflect changes in the level of brain function. Neurofeedback aids participants to voluntarily change brain function online using distinct cognitive strategies (e.g., focus on and away from drug-related stimuli). Neurofeedback has been most consistently tested in ADHD and other psychopathologies, with very early evidence being available in SUD.

Neurofeedback is a promising tool that enables mapping of the causal mechanisms of SUD. As core brain dysfunction is identified within a SUD, neurofeedback can be used as a personalized intervention to enhance and recover underlying dysfunctional neurocognitive pathways. Neurofeedback can source and target brain activity using distinct brain imaging techniques including EEG and fMRI (222).

EEG-based neurofeedback allows individuals to modulate the intensity of brain oscillations at specific frequencies (e.g., alpha, beta, theta, alpha-theta, theta-alpha). These protocols have often been used in conjunction with sensorimotor rhythm training (223) to improve efficacy in SUD. EEG-based neurofeedback studies have targeted brain function in varying SUD groups including alcohol, opioid, and stimulant use disorders [see detailed review here (224)]. This body of work led to mixed evidence of effects (and lack of) on abstinence in the week and months following neurofeedback training, as well as reduced disinhibition, craving, and severity of dependence symptoms. A paucity of studies has shown that these effects were stronger when EEG neurofeedback was used in conjunction with existing standard psychological, pharmacological, and rehabilitation treatments.

Real-time fMRI (rtfMRI)-based neurofeedback has the potential to provide insight in understanding the mechanisms of SUD underpinned by deep brain nuclei [e.g., striatum, amygdala (80)] the activity of which is unlikely to be robustly measured *via* surface EEG. Feedback can be provided on the level of activity of single or multiple *a priori* regions of interest, the strength of the connectivity between multiple regions, and patterns of brain activity identified with machine learning methods (e.g., support vector machine) (218). A handful of studies have used rtfMRI neurofeedback in SUD [for a review, see (12)]. This body of work focused largely on nicotine (225–230) and alcohol use disorders (231, 232).

Most of these studies focused on *a priori* brain regions of interest, most commonly the anterior cingulate cortex, medial prefrontal cortex, and other regions—as well as brain connectivity—were used as source for feedback from single studies (OFC, dorsomedial and dorsolateral prefrontal regions, insula and ventral striatum). Several neurofeedback studies required participants to modulate brain function during craving tasks (e.g., largely cue reactivity tasks that entail watching drug-related pictures). This body of work shows that patients could modulate brain function in the target regions, and provides mixed evidence on the presence and absence (226, 227, 229) of associations between changes in brain activity/connectivity and the severity of drug craving.

In EEG and rtfMRI neurofeedback studies, the significant lack of active placebo controlled and well-powered studies (e.g., comparison with a group with sham feedback) warrants the conduct of more systematic work to determine the efficacy of rEEG and rtfMRI-based neurofeedback.

### ISAM NIG Recommendations for Neuromodulation

As discussed above, there is a growing hope that neuromodulation can play a role in the daily practice of addiction medicine. However, the lack of rigorous designs does not provide strong enough evidence to give a green light for clinical use. With frequent negative trials for new pharmacological interventions in addiction medicine, governmental agencies across the world are seriously looking for new hopes for any intervention that can bring positive results in well-powered double-blinded sham/active controlled randomized trials. As the authors of this ISAM-NIG roadmap, we give the following recommendations for future work:

1. Creating international platforms that facilitate consensus on key targets for neuromodulation and outcome measures of efficacy: Addiction neuroscience suffers from the lack of international collaborations based on shared matrix of multilayer targets and outcome measures. We hope that ISAM NIG can bring together a critical mass of expert multidisciplinary scientists across the world to contribute in development of this international consensus.
2. Setting an agreed-upon minimum international standards to produce high quality evidence on the efficacy of neuromodulation in SUD: An overview on the scientific rigor in the published trials on tES/TMS for addiction medicine shows many methodological gaps (233). New potential solutions to address this may include shared protocols across labs internationally with leadership of expert scientists in the field, the development of quality control checklists and Delphi initiatives to reach a consensus on minimum standards.
3. Increase the power of neuromodulation experiments: Over 80% of tES/TMS/NFB studies reported 30 or less subjects in each of their arms. Sample sizes can be boosted using multi-site studies with shared protocols with or without shared funding and replication of previous and ongoing studies and trials across distinct laboratories. Larger samples will be instrumental to (i) increase the power to detect existing effects (or lack of), (ii) increase external validity (while accounting for inter-individual variability), (iii) make predictive modeling for responders and non-responders possible.
4. We also need to have studies with multi-session interventions and long term follow-up to examine the efficacy in tES/TMS/NF over time, particularly if it increases prolonged abstinence.
5. Strategize research efforts to focus available resources to examine the clinical feasibility/efficacy of neuromodulation: Huge parameter space in almost all areas of neuromodulation prevent providing high quality evidence necessary to inform clinical practice. Pharmaceutical companies are one of the main drivers of drug developments. There is no big company in the field of non-invasive neuromodulation and few new ones for TMS are still considered as "small businesses" (less than 250 employees). Efforts that pool sources of research support, e.g., targeted governmental funds and/or "crowd sourcing"-type collective international efforts may support the development and testing of harmonized neuromodulation protocols/target sites for intervention, in order to provide high quality, well-powered evidence.

## Conclusions

We reason that incorporating cognitive assessment into clinical practice in addiction treatment requires identification of constructs that predict meaningful clinical outcomes, streamlining of measures for clinical usability while improving retest reliability and ecological validity, and application of technology for remote monitoring and scalability. Translation of neuroimaging measures to clinically meaningful treatment outcomes requires developing imaging biomarkers that have mechanistic, diagnostic, and prognostic value. It also requires testing the cost-effectiveness of introducing brief, targeted brain scans, and deriving quantitative predictors of successful treatment outcome. Application of cognitive training/remediation and neuromodulation requires additional evidence from randomized trials and clear pathways to implementation. These translation efforts need to address all substance-related disorders. To date, most neuroscience studies have focused on alcohol, nicotine, cannabis, and stimulants, whereas opioids have been underrepresented. The promise of translational neuroscience will only be fulfilled if we can provide novel and effective solutions to pervasive addiction problems, for example, the current opioid crisis. Translation efforts should also factor in the heterogeneity of SUD populations in terms of principal drug of choice, patterns of polysubstance use and psychiatric comorbidities. In this regard, assessment and intervention protocols need to advance towards personalized approaches, by capitalizing on advanced machine learning applications.

**Figure 4:**
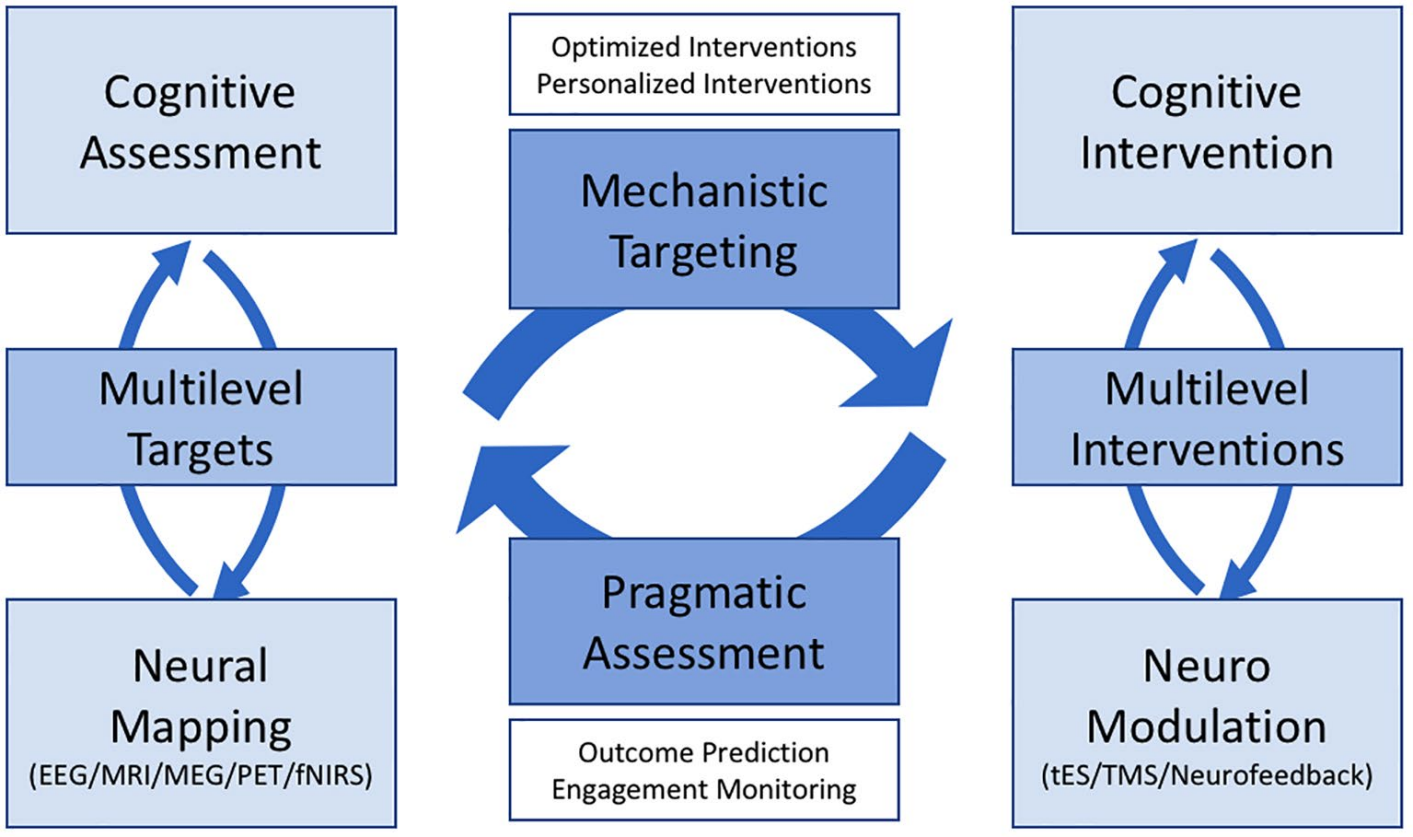
Neuroscience-informed addiction medicine in closed-loops. Cognitive assessments and different neural mapping technologies will introduce mechanistic targets (biomarkers) with neural/cognitive/behavioral levels for a combination of neuromodulation and cognitive interventions. Effects of interventions in successfully modifying these targets (biomarkers) are assessed with cognitive and neural mapping measures. Predictive models for treatment efficacy are optimized with Bayesian algorithms based on the pragmatic multilevel assessments. Interventions can be optimized in closed-loops to engage targets and consider personalized variations toward precision addiction medicine. Psychopharmacological interventions are not included in this roadmap paper; however, they could be delivered alongside and potentially augment cognitive training and neuromodulation.

Cognitive assessments and neuroimaging methods can elucidate mechanistic multi-level targets (biomarkers) with neural/cognitive/behavioral levels for neuroscience-informed individualized interventions (**Figure 4**). Neuromodulation and cognitive training interventions along with neuropharmacological agents could form multilevel adjunctive interventions based on these targets. The effects of these multilevel interventions in successfully targeting these mechanisms (biomarkers) should be assessed using cognitive and neural mapping measures. There remain many challenges to implementing neuroscience-informed addiction treatments. We propose to address these challenges by promoting international collaboration between researchers, clinicians, and industry, developing harmonized protocols and data collection/sharing platforms, and prioritizing research that focuses on improving clinical outcomes in SUD.

## Author Contributions

All authors have contributed in design and preparation of the manuscript. RH, RB, AV-G, VL, VM, DP, and HE created the first draft of assessment, imaging, training, and neuromodulation sections, respectively. AV-G and HE integrated all feedbacks from authors. All authors have agreed on the final manuscript before submission.

## Funding

AV-G is supported by Australian Medical Research Future Fund Fellowship (MRF1141214). DM is supported by NIH R01DA039903. MB receives support from NIH R01AA023667. DLP is supported by the Office of Research and Development, Clinical Science Research and Development (CSR&D), Department of Veterans Affairs, Career Development Award—2 (1IK2CX001510-01). SJM was supported by NIH K01DA037452.

## Conflict of Interest

AB has received research project funding from the MRC, CSO, Schering-Plough, Merck Serono, Lundbeck, and Indivior. ST has received unrestricted educational grants from Indivior, Lundbeck Foundation, and Merck Serono.

The remaining authors declare that the research was conducted in the absence of any commercial or financial relationships that could be construed as a potential conflict of interest.

## References

[1] Dominguez-Salas S Diaz-Batanero C Lozano-Rojas OM Verdejo-Garcia A Impact of general cognition and executive function deficits on addiction treatment outcomes: Systematic review and discussion of neurocognitive pathways. Neurosci Biobehav Rev (2016) 71:772–801. 10.1016/j.neubiorev.2016.09.030 27793597

[2] Ekhtiari H Victor TA Paulus MP Aberrant decision-making and drug addiction — how strong is the evidence? Curr Opin In Behav Sci (2017) 13:25–33. 10.1016/j.cobeha.2016.09.002

[3] Zilverstand A Huang AS Alia-Klein N Goldstein RZ Neuroimaging impaired response inhibition and salience attribution in human drug addiction: a systematic review. Neuron (2018) 98(5):886–903. 10.1016/j.neuron.2018.03.048 29879391 PMC5995133

[4] Passetti F Verdejo-García A Checinski K Robbins TW Bridging the gap between neurocognitive models and treatment in alcohol, opiates and stimulants addiction. Front Psychiatry (2014).

[5] Fernandez-Serrano MJ Perez-Garcia M Verdejo-Garcia A What are the specific vs. generalized effects of drugs of abuse on neuropsychological performance? Neurosci Biobehav Rev (2011) 35(3):377–406. 10.1016/j.neubiorev.2010.04.008 20451551

[6] Potvin S Pelletier J Grot S Hebert C Barr AM Lecomte T Cognitive deficits in individuals with methamphetamine use disorder: A meta-analysis. Addict Behav (2018) 80:154–60. 10.1016/j.addbeh.2018.01.021 29407687

[7] Potvin S Stavro K Rizkallah E Pelletier J Cocaine and cognition: a systematic quantitative review. J Addict Med (2014) 8(5):368–76. 10.1097/ADM.0000000000000066 25187977

[8] Rolland B D’Hondt F Montegue S Brion M Peyron E D’Aviau de Ternay J A patient-tailored evidence-based approach for developing early neuropsychological training programs in addiction settings. Neuropsychol Rev (2019) 29(1):103–15. 10.1007/s11065-018-9395-3 30607658

[9] Stavro K Pelletier J Potvin S Widespread and sustained cognitive deficits in alcoholism: a meta-analysis. Addict Biol (2013) 18(2):203–13. 10.1111/j.1369-1600.2011.00418.x 22264351

[10] Verdejo-Garcia A Neuroclinical assessment of addiction needs to incorporate decision-making measures and ecological validity. Biol Psychiatry (2017) 81(7):e53–4. 10.1016/j.biopsych.2016.07.015 27776736

[11] Kwako LE Momenan R Litten RZ Koob GF Goldman D Addictions neuroclinical assessment: a neuroscience-based framework for addictive disorders. Biol Psychiatry (2016) 80(3):179–89. 10.1016/j.biopsych.2015.10.024 PMC4870153 26772405

[12] Luigjes J Lorenzetti V de Haan S Youssef GJ Murawski C Sjoerds Z Defining compulsive behavior. Neuropsychol Rev (2019) 29(1):4–13. 10.1007/s11065-019-09404-9 31016439 PMC6499743

[13] Yucel M Oldenhof E Ahmed SH Belin D Billieux J Bowden-Jones H A transdiagnostic dimensional approach towards a neuropsychological assessment for addiction: an international Delphi consensus study. Addiction (2019) 114(6):1095–109. 10.1111/add.14424 PMC6386631 30133930

[14] Bates ME Buckman JF Nguyen TT A role for cognitive rehabilitation in increasing the effectiveness of treatment for alcohol use disorders. Neuropsychol Rev (2013) 23(1):27–47. 10.1007/s11065-013-9228-3 23412885 PMC3610413

[15] Schmidt P Haberthur A Soyka M Cognitive functioning in formerly opioid-dependent adults after atleast 1 year of abstinence: a naturalistic study. Eur Addict Res (2017) 23(6):269–75. 10.1159/000485032 29268271

[16] Vonmoos M Hulka LM Preller KH Minder F Baumgartner MR Quednow BB Cognitive impairment in cocaine users is drug-induced but partially reversible: evidence from a longitudinal study. Neuropsychopharmacology (2014) 39(9):2200–10. 10.1038/npp.2014.71 PMC4104339 24651468

[17] Rubenis AJ Fitzpatrick RE Lubman DI Verdejo-Garcia A Working memory predicts methamphetamine hair concentration over the course of treatment: moderating effect of impulsivity and implications for dual-systems model. Addict Biol (2019) 24(1):145–53. 10.1111/adb.12575 29114992

[18] Stevens L Verdejo-Garcia A Goudriaan AE Roeyers H Dom G Vanderplasschen W Impulsivity as a vulnerability factor for poor addiction treatment outcomes: a review of neurocognitive findings among individuals with substance use disorders. J Subst Abuse Treat (2014) 47(1):58–72. 10.1016/j.jsat.2014.01.008 24629886

[19] Rubenis AJ Fitzpatrick RE Lubman DI Verdejo-Garcia A Impulsivity predicts poorer improvement in quality of life during early treatment for people with methamphetamine dependence. Addiction (2018) 113(4):668–76. 10.1111/add.14058 28987070

[20] Tiffany ST Friedman L Greenfield SF Hasin DS Jackson R Beyond drug use: a systematic consideration of other outcomes in evaluations of treatments for substance use disorders. Addiction (2012) 107(4):709–18. 10.1111/j.1360-0443.2011.03581.x PMC3257402 21981638

[21] Roche A Pidd K Alcohol & Other Drugs Workforce Development Issues and Imperatives: Setting the Scene. Adelaide, National Centre for Education and Training on Addiction (NCETA), Flinders University (2010).

[22] ACT, A. T. O. D. A. (2016). Strengthening Specialist Alcohol and Other Drug Treatment and Support: Needsand Prioritiesforthe ACT 2016–2017. *A. T. O. D. A. ACT. Canberra, Alcohol Tobacco and Other Drug Association ACT.*

[23] Health Q Mental health alcohol and other drugs workforce development framework. Queensland State Queensland (Queensland Health) (2017).

[24] Roche A Kostadinov V Hodge S Duralsingham V McEntee A Pidd K Characteristics and wellbeing of the NSW non-government AOD Workforce. Adelaide, National Centre for Education and Training on Addiction, Flinders University (2018).

[25] Nelson A The SAGE Handbook of Drug and Alcohol Studies. SAGE Publications Ltd.: 55 City Road, London (2016). Retrieved from http://sk.sagepub.com/reference/the-sage-handbook-of-drug-alcohol-studies-v1. 10.4135/9781473921986

[26] Boustani M Peterson B Hanson L Harris R Lohr KN Force U S. P. S. T. Screening for dementia in primary care: a summary of the evidence for the U.S. Preventive Services Task Force. Ann Intern Med (2003) 138(11):927–37. 10.7326/0003-4819-138-11-200306030-00015 12779304

[27] Milne A Culverwell A Guss R Tuppen J Whelton R Screening for dementia in primary care: a review of the use, efficacy and quality of measures. Int Psychogeriatr (2008) 20(5):911–26. 10.1017/S1041610208007394 18533066

[28] Nuechterlein KH Green MF Kern RS Baade LE Barch DM Cohen JD The MATRICS Consensus Cognitive Battery, part 1: test selection, reliability, and validity. Am J Psychiatry (2008) 165(2):203–13. 10.1176/appi.ajp.2007.07010042 18172019

[29] Alarcon R Nalpas B Pelletier S Perney P MoCA as a Screening tool of neuropsychological deficits in alcohol-dependent patients. Alcohol Clin Exp Res (2015) 39(6):1042–8. 10.1111/acer.12734 25939560

[30] Copersino ML Fals-Stewart W Fitzmaurice G Schretlen DJ Sokoloff J Weiss RD Rapid cognitive screening of patients with substance use disorders. Exp Clin Psychopharmacol (2009) 17(5):337–44. 10.1037/a0017260 PMC3144764 19803633

[31] Copersino ML Schretlen DJ Fitzmaurice GM Lukas SE Faberman J Sokoloff J Effects of cognitive impairment on substance abuse treatment attendance: predictive validation of a brief cognitive screening measure. Am J Drug Alcohol Abuse (2012) 38(3):246–50. 10.3109/00952990.2012.670866 PMC3593077 22443860

[32] Manning V Staiger PK Hall K Garfield JB Flaks G Leung D Cognitive bias modification training during inpatient alcohol detoxification reduces early relapse: a randomized controlled trial. Alcohol Clin Exp Res (2016) 40(9):2011–9. 10.1111/acer.13163 27488392

[33] Manning V Wanigaratne S Best D Strathdee G Schrover I Gossop M Screening for cognitive functioning in psychiatric outpatients with schizophrenia, alcohol dependence, and dual diagnosis. Schizophr Res (2007) 91(1-3):151–8. 10.1016/j.schres.2006.11.019 17300919

[34] Ridley N Batchelor J Draper B Demirkol A Lintzeris N Withall A Cognitive screening in substance users: diagnostic accuracies of the mini-mental state examination, addenbrooke’s cognitive examination-revised, and montreal cognitive assessment. J Clin Exp Neuropsychol (2018) 40(2):107–22. 10.1080/13803395.2017.1316970 28436744

[35] Folstein MF Folstein SE McHugh PR "Mini-mental state". a practical method for grading the cognitive state of patients for the clinician. J Psychiatr Res (1975) 12(3):189–98. 10.1016/0022-3956(75)90026-6 1202204

[36] Mioshi E Dawson K Mitchell J Arnold R Hodges JR The Addenbrooke’s Cognitive Examination Revised (ACE-R): a brief cognitive test battery for dementia screening. Int J Geriatr Psychiatry (2006) 21(11):1078–85. 10.1002/gps.1610 16977673

[37] Nasreddine ZS Phillips NA Bedirian V Charbonneau S Whitehead V Collin I The montreal cognitive assessment, MoCA: a brief screening tool for mild cognitive impairment. J Am Geriatr Soc (2005) 53(4):695–9. 10.1111/j.1532-5415.2005.53221.x 15817019

[38] Manning V Teo HC Guo S Wong KE Li TK Neurocognitive functioning and treatment outcome following detoxification among asian alcohol-dependent inpatients. Subst Use Misuse (2016) 51(2):193–205. 10.3109/10826084.2015.1092985 26771240

[39] Kern RS Green MF Nuechterlein KH Deng BH NIMH-MATRICS survey on assessment of neurocognition in schizophrenia. Schizophr Res (2004) 72(1):11–9. 10.1016/j.schres.2004.09.004 15531403

[40] Kern RS Nuechterlein KH Green MF Baade LE Fenton WS Gold JM The MATRICS consensus cognitive battery, part 2: co-norming and standardization. Am J Psychiatry (2008) 165(2):214–20. 10.1176/appi.ajp.2007.07010043 18172018

[41] Conway KP Vullo GC Kennedy AP Finger MS Agrawal A Bjork JM Data compatibility in the addiction sciences: an examination of measure commonality. Drug Alcohol Depend (2014) 141:153–8. 10.1016/j.drugalcdep.2014.04.029 PMC4096981 24954640

[42] Robbins TW James M Owen AM Sahakian BJ Lawrence AD McInnes L A study of performance on tests from the CANTAB battery sensitive to frontal lobe dysfunction in a large sample of normal volunteers: implications for theories of executive functioning and cognitive aging. Cambridge Neuropsychological Test Automated Battery. J Int Neuropsychol Soc (1998) 4(5):474–90. 10.1017/s1355617798455073 9745237

[43] Randolph C Tierney MC Mohr E Chase TN The Repeatable Battery for the Assessment of Neuropsychological Status (RBANS): preliminary clinical validity. J Clin Exp Neuropsychol (1998) 20(3):310–9. 10.1076/jcen.20.3.310.823 9845158

[44] Stern RA White T NAB, Neuropsychological Assessment Battery: Administration, scoring, and interpretation manual. Psychological Assessment Resources Lutz (FL) (2003).

[45] Powell D Kaplan E Whitla D Weinstraub S Catlin R Funkenstein H MicroCog Assessment of cognitive functioning, windows^®^ edition (MicroCog™ for Windows®) San Antonio, TX. Psychol Corporation (2004).

[46] Cannizzaro DL Elliott JC Stohl M Hasin DS Aharonovich E Neuropsychological Assessment Battery-Screening Module (S-NAB): performance in treatment-seeking cocaine users. Am J Drug Alcohol Abuse (2014) 40(6):476–83. 10.3109/00952990.2014.916718 PMC4309379 24949996

[47] Latvala A Castaneda AE Perala J Saarni SI Aalto-Setala T Lonnqvist J Cognitive functioning in substance abuse and dependence: a population-based study of young adults. Addiction (2009) 104(9):1558–68. 10.1111/j.1360-0443.2009.02656.x 19686526

[48] Schrimsher GW Parker JD Changes in cognitive function during substance use disorder treatment. J Psychopathol Behav Assess (2008) 30(2):146–53. 10.1007/s10862-007-9054-0

[49] Bauer RM Iverson GL Cernich AN Binder LM Ruff RM Naugle RI Computerized neuropsychological assessment devices: joint position paper of the American Academy of Clinical Neuropsychology and the National Academy of Neuropsychology. Arch Clin Neuropsychol (2012) 27(3):362–73. 10.1093/arclin/acs027 PMC3499090 22382386

[50] Cole WR Arrieux JP Schwab K Ivins BJ Qashu FM Lewis SC Test-retest reliability of four computerized neurocognitive assessment tools in an active duty military population. Arch Clin Neuropsychol (2013) 28(7):732–42. 10.1093/arclin/act040 23819991

[51] Fratti S Bowden SC Cook MJ Reliability and validity of the CogState computerized battery in patients with seizure disorders and healthy young adults: comparison with standard neuropsychological tests. Clin Neuropsychol (2017) 31(3):569–86. 10.1080/13854046.2016.1256435 27852143

[52] Nelson LD LaRoche AA Pfaller AY Lerner EB Hammeke TA Randolph C Prospective, head-to-head study of three computerized Neurocognitive Assessment Tools (CNTs): reliability and validity for the assessment of sport-related concussion. J Int Neuropsychol Soc (2016) 22(1):24–37. 10.1017/S1355617715001101 26714883 PMC4882608

[53] Resch JE Schneider MW Munro Cullum C The test-retest reliability of three computerized neurocognitive tests used in the assessment of sport concussion. Int J Psychophysiol (2018) 132(Pt A):31–8. 10.1016/j.ijpsycho.2017.09.011 28935224

[54] Enkavi AZ Eisenberg IW Bissett PG Mazza GL MacKinnon DP Marsch LA Large-scale analysis of test-retest reliabilities of self-regulation measures. Proc Natl Acad Sci U.S.A. (2019) 116(12):5472–7. 10.1073/pnas.1818430116 PMC6431228 30842284

[55] Bouvard A Dupuy M Schweitzer P Revranche M Fatseas M Serre F Feasibility and validity of mobile cognitive testing in patients with substance use disorders and healthy controls. Am J Addict (2018) 27(7):553–6. 10.1111/ajad.12804 30260085

[56] Moore RC Swendsen J Depp CA Applications for self-administered mobile cognitive assessments in clinical research: a systematic review. Int J Methods Psychiatr Res (2017) 26(4):e1562. 10.1002/mpr.1562 PMC5623609 28370881

[57] Sliwinski MJ Mogle JA Hyun J Munoz E Smyth JM Lipton RB Reliability and validity of ambulatory cognitive assessments. Assessment (2018) 25(1):14–30. 10.1177/1073191116643164 27084835 PMC5690878

[58] Jones A McGrath E Robinson E Houben K Nederkoorn C Field M A randomized controlled trial of inhibitory control training for the reduction of alcohol consumption in problem drinkers. J Consult Clin Psychol (2018) 86(12):991–1004. 10.1037/ccp0000312 30507225 PMC6277130

[59] Crane D Garnett C Michie S West R Brown J A smartphone app to reduce excessive alcohol consumption: Identifying the effectiveness of intervention components in a factorial randomised control trial. Sci Rep (2018) 8(1):4384. 10.1038/s41598-018-22420-8 29531280 PMC5847600

[60] Jones A Tiplady B Houben K Nederkoorn C Field M Do daily fluctuations in inhibitory control predict alcohol consumption? An ecological momentary assessment study. Psychopharmacol (Berl) (2018) 235(5):1487–96. 10.1007/s00213-018-4860-5 PMC5919991 29497782

[61] Wiers CE Ludwig VU Gladwin TE Park SQ Heinz A Wiers RW Effects of cognitive bias modification training on neural signatures of alcohol approach tendencies in male alcohol-dependent patients. Addict Biol (2015) 20(5):990–9. 10.1111/adb.12221 25639749

[62] Eisenberg IW Bissett P Enkavi AZ Li J MacKinnon D Marsch L Uncovering mental structure through data-driven ontology discovery. PsyArXiv (2018). 10.31234/osf.io/fvqej PMC6534563 31127115

[63] Jones A Remmerswaal D Verveer I Robinson E Franken IHA Wen CKF Compliance with ecological momentary assessment protocols in substance users: a meta-analysis. Addiction (2019) 114(4):609–19. 10.1111/add.14503 PMC6492133 30461120

[64] Bos FM Schoevers RA aan het Rot M Experience sampling and ecological momentary assessment studies in psychopharmacology: a systematic review. Eur Neuropsychopharmacol (2015) 25(11):1853–64. 10.1016/j.euroneuro.2015.08.008 26336868

[65] Ramsey AT Wetherell JL Depp C Dixon D Lenze E Feasibility and acceptability of smartphone assessment in older adults with cognitive and emotional difficulties. J Technol Hum Serv (2016) 34(2):209–23. 10.1080/15228835.2016.1170649 PMC5036573 27683018

[66] Bickel WK Mellis AM Snider SE Athamneh LN Stein JS Pope DA 21st century neurobehavioral theories of decision making in addiction: review and evaluation. Pharmacol Biochem Behav (2018) 164:4–21. 10.1016/j.pbb.2017.09.009 28942119 PMC5747999

[67] Bates ME Bowden SC Barry D Neurocognitive impairment associated with alcohol use disorders: implications for treatment. Exp Clin Psychopharmacol (2002) 10(3):193–212. 10.1037//1064-1297.10.3.193 12233981

[68] Bates ME Buckman JF Voelbel GT Eddie D Freeman J The mean and the individual: integrating variable-centered and person-centered analyses of cognitive recovery in patients with substance use disorders. Front Psychiatry (2013) 4:177. 10.3389/fpsyt.2013.00177 24399976 PMC3870950

[69] Buelow MT Suhr JA Construct validity of the Iowa Gambling Task. Neuropsychol Rev (2009) 19(1):102–14. 10.1007/s11065-009-9083-4 19194801

[70] Field M Munafò MR Franken IH A meta-analytic investigation of the relationship between attentional bias and subjective craving in substance abuse. J Psychol Bull (2009) 135(4):589. 10.1037/a0015843 PMC2999821 19586163

[71] Grüsser SM Wrase J Klein S Hermann D Smolka MN Ruf M Cue-induced activation of the striatum and medial prefrontal cortex is associated with subsequent relapse in abstinent alcoholics. Psychopharmacology (2004) 175(3):296–302. 15127179 10.1007/s00213-004-1828-4

[72] Kosten T Scanley B Tucker K Oliveto A Prince C Sinha R Cue-induced brain activity changes and relapse in cocaine-dependent patients. Neuropsychopharmacology (2006) 31:644–50. 10.1038/sj.npp.1300851 16123763

[73] Huettel SA Song AW McCarthy G What is fMRI? In: Functional magnetic research imaging, 2nd ed, vol. 2 Sinauer: Sunderland, Massachusetts, USA (2009). p. 3–14.

[74] Leroy C Karila L Martinot JL Lukasiewicz M Duchesnay E Comtat C Striatal and extrastriatal dopamine transporter in cannabis and tobacco addiction: a high-resolution PET study. Addict Biol (2012) 17(6):981–90. 10.1111/j.1369-1600.2011.00356.x 21812871

[75] Volkow ND Ding YS Fowler JS Wang GJ Cocaine addiction: hypothesis derived from imaging studies with PET. J Addict Dis (1996) 15(4):55–71. 10.1300/J069v15n04_04 8943582

[76] Williams TM Davies SJ Taylor LG Daglish MR Hammers A Brooks DJ Brain opioid receptor binding in early abstinence from alcohol dependence and relationship to craving: an [11C]diprenorphine PET study. Eur Neuropsychopharmacol (2009) 19(10):740–8. 10.1016/j.euroneuro.2009.06.007 19595579

[77] Yoder KK Constantinescu CC Kareken DA Normandin MD Cheng TE O’Connor SJ Heterogeneous effects of alcohol on dopamine release in the striatum: a pet study. Alcohol Clin Exp Res (2007) 31(6):965–73. 10.1111/j.1530-0277.2007.00390.x 17428296

[78] Yeung AWK An updated survey on statistical thresholding and sample size of fMRI studies. Front Hum Neurosci (2018) 12:16. 10.3389/fnhum.2018.00016 eCollection 2018. 29434545 PMC5790797

[79] Nichols TE Das S Eickhoff SB Evans AC Glatard T Hanke M Best practices in data analysis and sharing in neuroimaging using MRI. Nat Neurosci (2017) 20(3):299–303. 10.1038/nn.4500 28230846 PMC5685169

[80] Koob GF Volkow ND Neurobiology of addiction: a neurocircuitry analysis. Lancet Psychiatry (2016) 3(8):760–73. 10.1016/S2215-0366(16)00104-8 PMC6135092 27475769

[81] Lewis M Brain change in addiction as learning, not disease. New Engl J Med (2018) 379(16):1551–60. 10.1056/NEJMra1602872 30332573

[82] Morris SE Cuthbert BN Research Domain Criteria: cognitive systems, neural circuits, and dimensions of behavior. Dialogues Clin Neurosci (2012) 14(1):29–37. 22577302 10.31887/DCNS.2012.14.1/smorris PMC3341647

[83] Paulus MP Stewart JL Interoception and drug addiction. Neuropharmacology (2014) 76 Pt B:342–50. 10.1016/j.neuropharm.2013.07.002 PMC3858461 23855999

[84] Volkow ND Fowler JS Wang GJ The addicted human brain viewed in the light of imaging studies: brain circuits and treatment strategies. Neuropharmacology (2004) 47 Suppl 1:3–13. 10.1016/j.neuropharm.2004.07.019 15464121

[85] Yücel M Oldenhof E Ahmed SH Belin D Billieux J Bowden-Jones H A transdiagnostic dimensional approach towards a neuropsychological assessment for addiction: an international Delphi consensus study. (2019). 114(6):1095–1109. 10.1111/add.14424 PMC6386631 30133930

[86] Verdejo-Garcia A Clark L Dunn BD The role of interoception in addiction: a critical review. Neurosci Biobehav Rev (2012) 36(8):1857–69. 10.1016/j.neubiorev.2012.05.007 22659642

[87] Oldham S Murawski C Fornito A Youssef G Yucel M Lorenzetti V The anticipation and outcome phases of reward and loss processing: A neuroimaging meta-analysis of the monetary incentive delay task. Hum Brain Mapp (2018) 39(8):3398–418. 10.1002/hbm.24184 PMC6055646 29696725

[88] Liu X Hairston J Schrier M Fan J Common and distinct networks underlying reward valence and processing stages: a meta-analysis of functional neuroimaging studies. Neurosci Biobehav Rev (2011) 35(5):1219–36. 10.1016/j.neubiorev.2010.12.012 PMC3395003 21185861

[89] Engelmann JM Versace F Robinson JD Minnix JA Lam CY Cui Y Neural substrates of smoking cue reactivity: a meta-analysis of fMRI studies. Neuroimage (2012) 60(1):252–62. 10.1016/j.neuroimage.2011.12.024 PMC3288122 22206965

[90] Schacht JP Anton RF Myrick H Functional neuroimaging studies of alcohol cue reactivity: a quantitative meta-analysis and systematic review. Addict Biol (2013) 18(1):121–33. 10.1111/j.1369-1600.2012.00464.x PMC3419322 22574861

[91] Lipszyc J Schachar R Inhibitory control and psychopathology: a meta-analysis of studies using the stop signal task. J Int Neuropsychol Soc (2010) 16(6):1064–76. 10.1017/S1355617710000895 20719043

[92] Simmonds DJ Pekar JJ Mostofsky SH Meta-analysis of Go/No-go tasks demonstrating that fMRI activation associated with response inhibition is task-dependent. Neuropsychologia (2008) 46(1):224–32. 10.1016/j.neuropsychologia.2007.07.015 PMC2327217 17850833

[93] Derrfuss J Brass M Neumann J von Cramon DY Involvement of the inferior frontal junction in cognitive control: meta-analyses of switching and Stroop studies. Hum Brain Mapp (2005) 25(1):22–34. 10.1002/hbm.20127 15846824 PMC6871679

[94] Schulz SM Neural correlates of heart-focused interoception: a functional magnetic resonance imaging meta-analysis. Philos Trans R Soc Lond B Biol Sci (2016) 371(1708):20160018. 10.1098/rstb.2016.0018 28080975 PMC5062106

[95] Stewart J Khalsa S Kuplicki R Paulus M . Interoceptive Dysfunction in Stimulant and Opioid Addiction. Biol Psychiatry (2019) 85(10):S210. 10.1016/j.biopsych.2019.03.529

[96] Paulus MP Feinstein JS Khalsa SS An active inference approach to interoceptive psychopathology. Annual Review of Clinical Psychology (2019) 15(1):97–122. 10.1146/annurev-clinpsy-050718-095617 PMC7280559 31067416

[97] Daughters SB Ross TJ Bell RP Yi JY Ryan J Stein EA Distress tolerance among substance users is associated with functional connectivity between prefrontal regions during a distress tolerance task. Addict Biol (2017) 22(5):1378–90. 10.1111/adb.12396 PMC5625840 27037525

[98] Jennifer YY Dichter GS Reese ED Bell RP Bartuska AD Stein JR Neural reward response to substance-free activity images in opiate use disorder patients with depressive symptoms. Drug Alcohol Dependence (2019) 198:180–89. 10.1016/j.drugalcdep.2019.01.047 30947052

[99] Garavan H Brennan K Hester R Whelan R The neurobiology of successful abstinence. Curr Opin In Neurobiol (2013) 23(4):668–74. 10.1016/j.conb.2013.01.029 PMC3706547 23510740

[100] Sofuoglu M Cognitive enhancement as a pharmacotherapy target for stimulant addiction. Addict (Abingdon England) (2010) 105(1):38–48. 10.1111/j.1360-0443.2009.02791.x PMC2808705 20078461

[101] Sofuoglu M DeVito EE Waters AJ Carroll KM Cognitive enhancement as a treatment for drug addictions. Neuropharmacology (2013) 64(1):452–63. 10.1016/j.neuropharm.2012.06.021 PMC3445733 22735770

[102] Zilverstand A Parvaz MA Moeller SJ Goldstein RZ Cognitive interventions for addiction medicine: Understanding the underlying neurobiological mechanisms. Prog Brain Res (2016) 224:285–304. 10.1016/bs.pbr.2015.07.019 26822363 PMC5206794

[103] Trabert W Betz T Niewald M Huber G Significant reversibility of alcoholic brain shrinkage within 3 weeks of abstinence. Acta Psychiatrica Scand (1995) 92(2):87–90. 10.1111/j.1600-0447.1995.tb09548.x 7572265

[104] Hanlon CA Dufault DL Wesley MJ Porrino LJ Elevated gray and white matter densities in cocaine abstainers compared to current users. Psychopharmacology (2011) 218(4):681–92. 10.1007/s00213-011-2360-y PMC3197798 21691942

[105] Kril J Halliday G Svoboda M Cartwright H The cerebral cortex is damaged in chronic alcoholics. Neuroscience (1997) 79(4):983–98. 10.1016/s0306-4522(97)00083-3 9219961

[106] Pfefferbaum A Sullivan E Mathalon D Shear P Rosenbloom M Lim KJAC Longitudinal changes in magnetic resonance imaging brain volumes in abstinent and relapsed alcoholics. Alcohol Clin Exp Res (1995) 19: (5):1177–91. 10.1111/j.1530-0277.1995.tb01598.x 8561288

[107] Roberts AJ Koob GF The neurobiology of addiction. Alcohol Health Res World (1997) 21(2):101–6. PMC6826825 15704343

[108] Stephens DN Duka T Cognitive and emotional consequences of binge drinking: role of amygdala and prefrontal cortex. Philos Trans R Soc B: Biol Sci (2008) 363(1507):3169–79. 10.1098/rstb.2008.0097 PMC2607328 18640918

[109] Bell RP Foxe JJ Nierenberg J Hoptman MJ Garavan H Assessing white matter integrity as a function of abstinence duration in former cocaine-dependent individuals. Drug Alcohol Depend (2011) 114(2-3):159–68. 10.1016/j.drugalcdep.2010.10.001 PMC3062648 21075564

[110] Gansler DA Harris GJ Oscar-Berman M Streeter C Lewis RF Ahmed I Hypoperfusion of inferior frontal brain regions in abstinent alcoholics: a pilot SPECT study. J Stud Alcohol (2000) 61(1):32–7. 10.15288/jsa.2000.61.32 10627094

[111] Connolly CG Foxe JJ Nierenberg J Shpaner M Garavan H The neurobiology of cognitive control in successful cocaine abstinence. Drug Alcohol Depend (2012) 121(1-2):45–53. 21885214 10.1016/j.drugalcdep.2011.08.007 PMC3262906

[112] Nestor L McCabe E Jones J Clancy L Garavan H Differences in "bottom-up" and "top-down" neural activity in current and former cigarette smokers: evidence for neural substrates which may promote nicotine abstinence through increased cognitive control. Neuroimage (2011) 56(4):2258–75. 10.1016/j.neuroimage.2011.03.054 21440645

[113] Tapert SF Schweinsburg AD Drummond SP Paulus MP Brown SA Yang TT Functional MRI of inhibitory processing in abstinent adolescent marijuana users. Psychopharmacology (2007) 194(2):173–83. 10.1007/s00213-007-0823-y PMC2269705 17558500

[114] Gazdzinski S Durazzo TC Meyerhoff DJ Temporal dynamics and determinants of whole brain tissue volume changes during recovery from alcohol dependence. Drug Alcohol Depend (2005) 78(3):263–73. 10.1016/j.drugalcdep.2004.11.004 15893157

[115] Bolla K Ernst M Kiehl K Mouratidis M Eldreth D Contoreggi C Prefrontal cortical dysfunction in abstinent cocaine abusers. J Neuropsychiatry Clin Neurosci (2004) 16(4):456–64. 10.1176/appi.neuropsych.16.4.456 PMC2771441 15616172

[116] Froeliger B Kozink RV Rose JE Behm FM Salley AN McClernon FJ Hippocampal and striatal gray matter volume are associated with a smoking cessation treatment outcome: results of an exploratory voxel-based morphometric analysis. Psychopharmacology (2010) 210(4):577–83. 10.1007/s00213-010-1862-3 20424827

[117] Janes AC Pizzagalli DA Richardt S Chuzi S Pachas G Culhane MA Brain reactivity to smoking cues prior to smoking cessation predicts ability to maintain tobacco abstinence. Biol Psychiatry (2010) 67(8):722–9. 10.1016/j.biopsych.2009.12.034 PMC2954596 20172508

[118] Rando K Hong K-I Bhagwagar Z Li C-SR Bergquist K Guarnaccia J Association of frontal and posterior cortical gray matter volume with time to alcohol relapse: a prospective study. Am J Psychiatry (2011) 168(2):183–92. 10.1176/appi.ajp.2010.10020233 PMC3668974 21078704

[119] Sorg SF Taylor MJ Alhassoon OM Gongvatana A Theilmann RJ Frank LR Frontal white matter integrity predictors of adult alcohol treatment outcome. Biol Psychiatry (2012) 71(3):262–8. 10.1016/j.biopsych.2011.09.022 PMC4208753 22047719

[120] Brewer JA Worhunsky PD Carroll KM Rounsaville BJ Potenza MN Pretreatment brain activation during stroop task is associated with outcomes in cocaine-dependent patients. Biol Psychiatry (2008) 64(11):998–1004. 18635157 10.1016/j.biopsych.2008.05.024 PMC2601637

[121] Clark V Beatty G Anderson R Kodituwakku P Phillips J Lane T Reduced fMRI activity predicts relapse in patients recovering from stimulant dependence. Hum Brain Mapp (2014) 35(2):414–28. 10.1002/hbm.22184 PMC4470394 23015512

[122] Heinz A Wrase J Kahnt T Beck A Bromand Z Grüsser SM Brain activation elicited by affectively positive stimuli is associated with a lower risk of relapse in detoxified alcoholic subjects. Alcohol: Clin Exp Res (2007) 31(7):1138–47. 10.1111/j.1530-0277.2007.00406.x 17488322

[123] Jia Z Worhunsky PD Carroll KM Rounsaville BJ Stevens MC Pearlson GD An initial study of neural responses to monetary incentives as related to treatment outcome in cocaine dependence. Biol Psychiatry (2011) 70: (6):553–60. 10.1016/j.biopsych.2011.05.008 PMC3162064 21704307

[124] Paulus M Tapert S Schuckit M Neural activation patterns of methamphetamine-dependent subjects during decision making predict relapse. Arch Gen Psychiatry (2005) 62(7):761–8. 10.1001/archpsyc.62.7.761 15997017

[125] Durazzo T Pathak V Gazdzinski S Mon A Meyerhoff D Metabolite levels in the brain reward pathway discriminate those who remain abstinent from those who resume hazardous alcohol consumption after treatment for alcohol dependence. J Stud Alcohol Drugs (2010) 71(2):278–89. 10.15288/jsad.2010.71.278 PMC2841738 20230726

[126] Noel X Sferrazza R Van der Linden M Paternot J Verhas M Hanak C Contribution of frontal cerebral blood flow measured by Tc-99m-bicisate spect and executive function deficits to predicting treatment outcome in alcohol-dependent patients. Alcohol (2002) 37(4):347–54. 10.1093/alcalc/37.4.347 12107037

[127] Wang GJ Smith L Volkow ND Telang F Logan J Tomasi D Decreased dopamine activity predicts relapse in methamphetamine abusers. Mol Psychiatry (2012) 17(9):918. 21747399 10.1038/mp.2011.86 PMC3261322

[128] Verdejo-Garcia A Cognitive training for substance use disorders: Neuroscientific mechanisms. Neurosci Biobehav Rev (2016) 68:270–81. 10.1016/j.neubiorev.2016.05.018 27236041

[129] Field M Duka T Tyler E Schoenmakers T Attentional bias modification in tobacco smokers. J Nicotine Tobacco Res (2009) 11(7):812–22. 10.1093/ntr/ntp067 19474181

[130] Wiers CE Wiers RW Imaging the neural effects of cognitive bias modification training. Neuroimage (2017) 151:81–91. 10.1016/j.neuroimage.2016.07.041 27450074

[131] Volkow ND Swanson JM Evins AE DeLisi LE Meier MH Gonzalez R Effects of cannabis use on human behavior, including cognition, motivation, and psychosis: a review. JAMA Psychiatry (2016) 73(3):292–7. 10.1001/jamapsychiatry.2015.3278 26842658

[132] Chen AJ Novakovic-Agopian T Nycum TJ Song S Turner GR Hills NK Training of goal-directed attention regulation enhances control over neural processing for individuals with brain injury. Brain (2011) 134(Pt 5):1541–54. 10.1093/brain/awr067 PMC6410956 21515904

[133] Garland EL Howard MO Mindfulness-based treatment of addiction: current state of the field and envisioning the next wave of research. Addict Sci Clin Pract (2018) 13(1):14. 10.1186/s13722-018-0115-3 29669599 PMC5907295

[134] Anicha CL Ode S Moeller SK Robinson M Toward a cognitive view of trait mindfulness: distinct cognitive skills predict its observing and nonreactivity facets. J Pers (2012) 80(2):255–85. 10.1111/j.1467-6494.2011.00722.x 21299556

[135] Li W Howard MO Garland EL McGovern P Lazar M Mindfulness treatment for substance misuse: A systematic review and meta-analysis. J Subst Abuse Treat (2017) 75:62–96. 10.1016/j.jsat.2017.01.008 28153483

[136] Brewer JA Elwafi HM Davis JH Craving to Quit: psychological models and neurobiological mechanisms of mindfulness training as treatment for addictions. Psychol Addict Behav (2013) 27(2):366–79. 10.1037/a0028490 PMC3434285 22642859

[137] Froeliger B Mathew AR McConnell P Eichberg C Saladin M Carpenter M Restructuring reward mechanisms in nicotine addiction: a pilot fMRI study of mindfulness-oriented recovery enhancement for cigarette smokers. J Evidence-Based Complementary Altern Med (2017). 10.1155/2017/7018014 PMC5360937 28373890

[138] Calhoun V Adali T (2006). “Fusion of Multisubject Hemodynamic and Event-Related Potential Data Using Independent Component Analysis.,” in Paper presented at the 2006 IEEE International Conference on Acoustics Speech and Signal Processing Proceedings. (Vol. 5, pp. V-V). IEEE.

[139] Picton TW Electrophysiology of Mind: Event-Related Brain Potentials and Cognition. Vol. 33 Rugg MD Coles MGH Oxford University Press: Oxford, England (1996) p. 612–3 1995 *Psychophysiology*. 10.1111/j.1469-8986.1996.tb02439.x

[140] Campanella S Schroder E Kajosch H Noel X Kornreich C Why cognitive event-related potentials (ERPs) should have a role in the management of alcohol disorders. Neurosci Biobehav Rev (2018). 106:234–244 10.1016/j.neubiorev.2018.06.016 29936112

[141] Mackey S Kan KJ Chaarani B Alia-Klein N Batalla A Brooks S Genetic imaging consortium for addiction medicine: From neuroimaging to genes. Prog Brain Res (2016) 224:203–23. 10.1016/bs.pbr.2015.07.026 PMC4820288 26822360

[142] Gorey C Kuhns L Smaragdi E Kroon E Cousijn J Age-related differences in the impact of cannabis use on the brain and cognition: a systematic review. Eur Arch Psychiatry Clin Neurosci (2019) 1–22. 10.1007/s00406-019-00981-7 PMC6394430 30680487

[143] Scott JC Rosen AF Moore TM Roalf DR Satterthwaite TD Calkins ME Cannabis use in youth is associated with limited alterations in brain structure. Neuropsychopharmacology (2019) 1:1362–69. 10.1038/s41386-019-0347-2 PMC6784999 30780151

[144] Scott JC Wolf DH Calkins ME Bach EC Weidner J Ruparel K Cognitive functioning of adolescent and young adult cannabis users in the Philadelphia Neurodevelopmental Cohort. Psychol Addictive Behav (2017) 31(4):423. 10.1037/adb0000268 PMC5468477 28414475

[145] Fleury MJ Djouini A Huynh C Tremblay J Ferland F Menard JM Remission from substance use disorders: A systematic review and meta-analysis. Drug Alcohol Depend (2016) 168:293–306. 10.1016/j.drugalcdep.2016.08.625 27614380

[146] Rezapour T DeVito EE Sofuoglu M Ekhtiari H Perspectives on neurocognitive rehabilitation as an adjunct treatment for addictive disorders: from cognitive improvement to relapse prevention. Prog Brain Res (2016) 224:345–69. 10.1016/bs.pbr.2015.07.022 26822366

[147] Boffo M Zerhouni O Gronau QF van Beek RJJ Nikolaou K Marsman M Cognitive bias modification for behavior change in alcohol and smoking addiction: bayesian meta-analysis of individual participant data. Neuropsychol Rev (2019) 29(1):52–78. 10.1007/s11065-018-9386-4 30644025 PMC6499757

[148] Eack SM Hogarty SS Bangalore SS Keshavan MS Cornelius JR Patterns of substance use during cognitive enhancement therapy: an 18-month randomized feasibility study. J Dual Diagn (2016) 12(1):74–82. 10.1080/15504263.2016.1145778 27089154 PMC4837677

[149] Eack SM Hogarty SS Greenwald DP Litschge MY McKnight SA Bangalore SS Cognitive Enhancement Therapy in substance misusing schizophrenia: results of an 18-month feasibility trial. Schizophr Res (2015) 161(2-3):478–83. 10.1016/j.schres.2014.11.017 PMC4308498 25510926

[150] Rezapour T Hatami J Farhoudian A Sofuoglu M Noroozi A Daneshmand R Cognitive rehabilitation for individuals with opioid use disorder: a randomized controlled trial. Neuropsychol Rehabil (2019) 29(8):1273–89. 10.1080/09602011.2017.1391103 29161998

[151] Rupp CI Kemmler G Kurz M Hinterhuber H Fleischhacker WW Cognitive remediation therapy during treatment for alcohol dependence. J Stud Alcohol Drugs (2012) 73(4):625–34. 10.15288/jsad.2012.73.625 22630801

[152] Bell MD Laws HB Petrakis IB A randomized controlled trial of cognitive remediation and work therapy in the early phase of substance use disorder recovery for older veterans: neurocognitive and substance use outcomes. Psychiatr Rehabil J (2017) 40(1):94–102. 10.1037/prj0000211 27732034 PMC5378626

[153] Bell MD Vissicchio NA Weinstein AJ Cognitive training and work therapy for the treatment of verbal learning and memory deficits in veterans with alcohol use disorders. J Dual Diagn (2016) 12(1):83–9. 10.1080/15504263.2016.1145779 PMC4932894 26828571

[154] Rezapour T Wurfel B Simblett S Ekhtiari H Neuropsychological Rehabilitation for Psychiatric Disorders. In: Neuropsychological Rehabilitation: The International Handbook, Routledge, New York vol. 136 (2017).

[155] Wojtalik JA Hogarty SS Cornelius JR Phillips ML Keshavan MS Newhill CE Cognitive Enhancement therapy improves frontolimbic regulation of emotion in alcohol and/or cannabis misusing schizophrenia: a preliminary study. Front Psychiatry (2015) 6:186. 10.3389/fpsyt.2015.00186 26793128 PMC4709416

[156] Labs L (2019). Lumosity. Retrieved from https://www.lumosity.com/.

[157] Marceau EM Berry J Lunn J Kelly PJ Solowij N Cognitive remediation improves executive functions, self-regulation and quality of life in residents of a substance use disorder therapeutic community. Drug Alcohol Depend (2017) 178:150–8. 10.1016/j.drugalcdep.2017.04.023 28651150

[158] Alfonso JP Caracuel A Delgado-Pastor LC Verdejo-Garcia A Combined Goal management training and mindfulness meditation improve executive functions and decision-making performance in abstinent polysubstance abusers. Drug Alcohol Depend (2011) 117(1):78–81. 10.1016/j.drugalcdep.2010.12.025 21277705

[159] Valls-Serrano C Caracuel A Verdejo-Garcia A Goal Management Training and Mindfulness Meditation improve executive functions and transfer to ecological tasks of daily life in polysubstance users enrolled in therapeutic community treatment. Drug Alcohol Depend (2016) 165:9–14. 10.1016/j.drugalcdep.2016.04.040 27246405

[160] Stamenova V Levine B Effectiveness of goal management training(R) in improving executive functions: A meta-analysis. Neuropsychol Rehabil (2018) 29(10):1569–99. 10.1080/09602011.2018.1438294 29540124

[161] Cogmed I (2019). Cogmed. Retrieved from http://www.cogmed.com.au/.

[162] Services PS (2014). PSSCogRehab. Retrieved from http://www.psychological-software.com/psscogrehab.html.

[163] Houben K Nederkoorn C Wiers RW Jansen A Resisting temptation: decreasing alcohol-related affect and drinking behavior by training response inhibition. Drug Alcohol Depend (2011) 116(1–3):132–6. 10.1016/j.drugalcdep.2010.12.011 21288663

[164] Wanmaker S Leijdesdorff SMJ Geraerts E de Wetering BJM Renkema PJ Franken IHA The efficacy of a working memory training in substance use patients: A randomized double-blind placebo-controlled clinical trial. J Clin Exp Neuropsychol (2018) 40(5):473–86. 10.1080/13803395.2017.1372367 28933254

[165] Lechner WV Sidhu NK Kittaneh AA Anand A Interventions with potential to target executive function deficits in addiction: current state of the literature. Curr Opin Psychol (2019) 30:24–8. 10.1016/j.copsyc.2019.01.017 30797130

[166] Khemiri L Brynte C Stunkel A Klingberg T Jayaram-Lindstrom N Working memory training in alcohol use disorder: a randomized controlled trial. Alcohol Clin Exp Res (2019) 43(1):135–46. 10.1111/acer.13910 PMC6587824 30462837

[167] Snider SE Deshpande HU Lisinski JM Koffarnus MN LaConte SM Bickel WK Working memory training improves alcohol users’ episodic future thinking: a rate-dependent analysis. Biol Psychiatry Cognit Neurosci Neuroimaging (2018) 3(2):160–7. 10.1016/j.bpsc.2017.11.002 PMC5851289 29529411

[168] Rass O Schacht RL Buckheit K Johnson MW Strain EC Mintzer MZ A randomized controlled trial of the effects of working memory training in methadone maintenance patients. Drug Alcohol Depend (2015) 156:38–46. 10.1016/j.drugalcdep.2015.08.012 26404954 PMC4633307

[169] Sweeney MM Rass O DiClemente C Schacht RL Vo HT Fishman MJ Working memory training for adolescents with cannabis use disorders: a randomized controlled trial. J Child Adolesc Subst Abuse (2018) 27(4):211–26. 10.1080/1067828X.2018.1451793 PMC6277048 30524179

[170] Brooks SJ Wiemerslage L Burch KH Maiorana SA Cocolas E Schioth HB The impact of cognitive training in substance use disorder: the effect of working memory training on impulse control in methamphetamine users. Psychopharmacol (Berl) (2017) 234(12):1911–21. 10.1007/s00213-017-4597-6 PMC5486910 28324119

[171] Houben K Wiers RW Response inhibition moderates the relationship between implicit associations and drinking behavior. Alcohol Clin Exp Res (2009) 33(4):626–33. 10.1111/j.1530-0277.2008.00877.x 19183132

[172] Luijten M Machielsen MW Veltman DJ Hester R de Haan L Franken IH Systematic review of ERP and fMRI studies investigating inhibitory control and error processing in people with substance dependence and behavioural addictions. J Psychiatry Neurosci (2014) 39(3):149–69. 10.1503/jpn.130052 PMC3997601 24359877

[173] Murphy P Garavan H Cognitive predictors of problem drinking and AUDIT scores among college students. Drug Alcohol Depend (2011) 115(1-2):94–100. 10.1016/j.drugalcdep.2010.10.011 21145183

[174] Verdejo-Garcia A Lawrence AJ Clark L Impulsivity as a vulnerability marker for substance-use disorders: review of findings from high-risk research, problem gamblers and genetic association studies. Neurosci Biobehav Rev (2008) 32(4):777–810. 10.1016/j.neubiorev.2007.11.003 18295884

[175] Houben K Wiers RW Jansen A Getting a grip on drinking behavior: training working memory to reduce alcohol abuse. Psychol Sci (2011) 22(7):968–75. 10.1177/0956797611412392 21685380

[176] Di Lemma LCG Field M Cue avoidance training and inhibitory control training for the reduction of alcohol consumption: a comparison of effectiveness and investigation of their mechanisms of action. Psychopharmacol (Berl) (2017) 234(16):2489–98. 10.1007/s00213-017-4639-0 PMC5537323 28551714

[177] Kilwein TM Bernhardt KA Stryker ML Looby A Decreased alcohol consumption after pairing alcohol-related cues with an inhibitory response. J Subst Use (2018) 23(2):154–61. 10.1080/14659891.2017.1378736

[178] Allom V Mullan B Hagger M Does inhibitory control training improve health behaviour? A meta-analysis. Health Psychol Rev (2016) 10(2):168–86. 10.1080/17437199.2015.1051078 26058688

[179] Jones A Di Lemma LC Robinson E Christiansen P Nolan S Tudur-Smith C Inhibitory control training for appetitive behaviour change: A meta-analytic investigation of mechanisms of action and moderators of effectiveness. Appetite (2016) 97:16–28. 10.1016/j.appet.2015.11.013 26592707

[180] Houben K Havermans RC Nederkoorn C Jansen A Beer a no-go: learning to stop responding to alcohol cues reduces alcohol intake *via* reduced affective associations rather than increased response inhibition. Addiction (2012) 107(7):1280–7. 10.1111/j.1360-0443.2012.03827.x 22296168

[181] Strickland JC Hill JC Stoops WW Rush CR Feasibility, acceptability, and initial efficacy of delivering alcohol use cognitive interventions *via* crowdsourcing. Alcohol Clin Exp Res (2019) 43(5):888–99. 10.1111/acer.13987 30888705

[182] Christiansen P Schoenmakers TM Field M Less than meets the eye: reappraising the clinical relevance of attentional bias in addiction. Addict Behav (2015) 44:43–50. 10.1016/j.addbeh.2014.10.005 25453782

[183] Fadardi JS Cox WM Reversing the sequence: reducing alcohol consumption by overcoming alcohol attentional bias. Drug Alcohol Depend (2009) 101(3):137–45. 10.1016/j.drugalcdep.2008.11.015 19193499

[184] McGeary JE Meadows SP Amir N Gibb BE Computer-delivered, home-based, attentional retraining reduces drinking behavior in heavy drinkers. Psychol Addict Behav (2014) 28(2):559–62. 10.1037/a0036086 PMC4068274 24955674

[185] Schoenmakers TM de Bruin M Lux IF Goertz AG Van Kerkhof DH Wiers RW Clinical effectiveness of attentional bias modification training in abstinent alcoholic patients. Drug Alcohol Depend (2010) 109(1–3):30–6. 10.1016/j.drugalcdep.2009.11.022 20064698

[186] Rinck M Wiers RW Becker ES Lindenmeyer J Relapse prevention in abstinent alcoholics by cognitive bias modification: Clinical effects of combining approach bias modification and attention bias modification. J Consult Clin Psychol (2018) 86(12):1005–16. 10.1037/ccp0000321 30507226

[187] Ziaee SS Fadardi JS Cox WM Yazdi SA Effects of attention control training on drug abusers’ attentional bias and treatment outcome. J Consult Clin Psychol (2016) 84(10):861–73. 10.1037/a0040290 27281374

[188] Mayer AR Wilcox CE Dodd AB Klimaj SD Dekonenko CJ Claus ED The efficacy of attention bias modification therapy in cocaine use disorders. Am J Drug Alcohol Abuse (2016) 42(4):459–68. 10.3109/00952990.2016.1151523 PMC4979538 27184297

[189] Dean AC Nurmi EL Moeller SJ Amir N Rozenman M Ghahremani DG No effect of attentional bias modification training in methamphetamine users receiving residential treatment. Psychopharmacol (Berl) (2019) 236(2):709–21. 10.1007/s00213-018-5100-8 PMC6415773 30415277

[190] Heitmann J Bennik EC van Hemel-Ruiter ME de Jong PJ The effectiveness of attentional bias modification for substance use disorder symptoms in adults: a systematic review. Syst Rev (2018) 7(1):160. 10.1186/s13643-018-0822-6 30316302 PMC6186103

[191] Eberl C Wiers RW Pawelczack S Rinck M Becker ES Lindenmeyer J Approach bias modification in alcohol dependence: do clinical effects replicate and for whom does it work best? Dev Cognit Neurosci (2013) 4:38–51. 10.1016/j.dcn.2012.11.002 23218805 PMC6987692

[192] Wiers RW Eberl C Rinck M Becker ES Lindenmeyer J Retraining automatic action tendencies changes alcoholic patients’ approach bias for alcohol and improves treatment outcome. Psychol Sci (2011) 22(4):490–7. 10.1177/0956797611400615 21389338

[193] Wiers RW Boffo M Field M What’s in a Trial? on the importance of distinguishing between experimental lab studies and randomized controlled trials: the case of cognitive bias modification and alcohol use disorders. J Stud Alcohol Drugs (2018) 79(3):333–43. 10.15288/jsad.2018.79.333 29885138

[194] Mann K Hoch E Batra A Bonnet U Gunthner A Reymann G Guideline-oriented treatment of alcohol-related disorders. Nervenarzt (2016) 87(1):13–25. 10.1007/s00115-015-0022-8 26670021

[195] Wiers CE Stelzel C Gladwin TE Park SQ Pawelczack S Gawron CK Effects of cognitive bias modification training on neural alcohol cue reactivity in alcohol dependence. Am J Psychiatry (2015) 172(4):335–43. 10.1176/appi.ajp.2014.13111495 25526597

[196] Sherman BJ Baker NL Squeglia LM Rae-Clark AL Approach bias modification for cannabis use disorder: a proof-of-principle study. J Subst Abuse Treat (2018) 87:16–22. 10.1016/j.jsat.2018.01.012 29471922 PMC5826579

[197] Kakoschke N Kemps E Tiggemann M Approach bias modification training and consumption: A review of the literature. Addict Behav (2017) 64:21–8. 10.1016/j.addbeh.2016.08.007 27538198

[198] Cristea IA Kok RN Cuijpers P The effectiveness of cognitive bias modification interventions for substance addictions: a meta-analysis. PloS One (2016) 11(9):e0162226. 10.1371/journal.pone.0162226 27611692 PMC5017662

[199] Zhu Y Jiang H Su H Zhong N Li R Li X A newly designed mobile-based computerized cognitive addiction therapy app for the improvement of cognition impairments and risk decision making in methamphetamine use disorder: randomized controlled trial. JMIR Mhealth Uhealth (2018) 6(6):e10292. 10.2196/10292 29925497 PMC6031898

[200] Kakoschke N Kemps E Tiggemann M What is the appropriate control condition for approach bias modification? A response to commentary by Becker et al. (2017). Addict Behav (2018) 77:295–6. 10.1016/j.addbeh.2017.02.024 28238576

[201] Lindgren KP Neighbors C Teachman BA Wiers RW Westgate E Greenwald AG I drink therefore I am: validating alcohol-related implicit association tests. Psychol Addict Behav (2013) 27(1):1–13. 10.1037/a0027640 22428863 PMC3604126

[202] Peeters M Wiers RW Monshouwer K de Schoot R Janssen T Vollebergh WA Automatic processes in at-risk adolescents: the role of alcohol-approach tendencies and response inhibition in drinking behavior. Addiction (2012) 107(11):1939–46. 10.1111/j.1360-0443.2012.03948.x 22632107

[203] Ekhtiari H Faghiri A Oghabian MA Paulus MP Functional neuroimaging for addiction medicine: From mechanisms to practical considerations. Prog Brain Res (2016) 224:129–53. 10.1016/bs.pbr.2015.10.001 26822357

[204] Ekhtiari H Nasseri P Yavari F Mokri A Monterosso J Neuroscience of drug craving for addiction medicine: from circuits to therapies. Prog Brain Res (2016) 223:115–41. 10.1016/bs.pbr.2015.10.002 26806774

[205] Ekhtiari H Paulus M Preface: Neuroscience for addiction medicine: from prevention to rehabilitation. Prog Brain Res (2016) 224:xxv–xxvi. 10.1016/s0079-6123(16)00030-3 26822370

[206] Parkin BL Ekhtiari H Walsh VF Non-invasive human brain stimulation in cognitive neuroscience: a primer. Neuron (2015) 87(5):932–45. 10.1016/j.neuron.2015.07.032 26335641

[207] Kwako LE Schwandt ML Ramchandani VA Diazgranados N Koob GF Volkow ND Neurofunctional domains derived from deep behavioral phenotyping in alcohol use disorder. Am J Psychiatry (2019) 176(9):744–53 10.1176/appi.ajp.2018.18030357 PMC6609498 30606047

[208] Yavari F Shahbabaie A Leite J 12(3):606-618 valho S Ekhtiari H Fregni F Noninvasive brain stimulation for addiction medicine: From monitoring to modulation. Prog Brain Res (2016) 224:371–99. 10.1016/bs.pbr.2015.08.007 26822367

[209] Jansen JM Daams JG Koeter MW Veltman DJ van den Brink W Goudriaan AE Effects of non-invasive neurostimulation on craving: a meta-analysis. Neurosci Biobehav Rev (2013) 37(10):2472–80. 10.1016/j.neubiorev.2013.07.009 23916527

[210] Maiti R Mishra BR Hota D Effect of high-frequency transcranial magnetic stimulation on craving in substance use disorder: a meta-analysis. J Neuropsychiatry Clin Neurosci (2016) 29(2):160–71. 10.1176/appi.neuropsych.16040065 27707195

[211] Song S Zilverstand A Gui W Li HJ Zhou X Effects of single-session versus multi-session non-invasive brain stimulation on craving and consumption in individuals with drug addiction, eating disorders or obesity: A meta-analysis. Brain Stimulation (2018) 12(3):606–18. 10.1016/j.brs.2018.12.975 30612944

[212] Kang N Kim RK Kim HJ Effects of transcranial direct current stimulation on symptoms of nicotine dependence: a systematic review and meta-analysis. Addictive Behav (2019) 96:133–9. 10.1016/j.addbeh.2019.05.006 31078740

[213] Terraneo A Leggio L Saladini M Ermani M Bonci A Gallimberti L Transcranial magnetic stimulation of dorsolateral prefrontal cortex reduces cocaine use: a pilot study. Eur Neuropsychopharmacol (2016) 26(1):37–44. 10.1016/j.euroneuro.2015.11.011 26655188 PMC9379076

[214] Yang LZ Shi B Li H Zhang W Liu Y Wang H Electrical stimulation reduces smokers’ craving by modulating the coupling between dorsal lateral prefrontal cortex and parahippocampal gyrus. Soc Cognit Affect Neurosci (2017) 12(8):1296–302. 10.1093/scan/nsx055 PMC5597850 28398588

[215] Kearney-Ramos TE Dowdle LT Lench DH Mithoefer OJ Devries WH George MS Transdiagnostic effects of ventromedial prefrontal cortex transcranial magnetic stimulation on cue reactivity. Biol Psychiatry Cognit Neurosci Neuroimaging (2018) 3(7):599–609. 10.1016/j.bpsc.2018.03.016 29776789 PMC6641556

[216] Krystal AD Pizzagalli DA Mathew SJ Sanacora G Keefe R Song A The first implementation of the NIMH FAST-FAIL approach to psychiatric drug development. Nat Rev Drug Discovery (2018) 18(1):82–4. 10.1038/nrd.2018.222 PMC6816017 30591715

[217] Lorenz R Simmons LE Monti RP Arthur JL Limal S Laakso I Assessing tACS-induced phosphene perception using closed-loop Bayesian optimization. bioRxiv (2017), 150086. 10.1101/150086

[218] Lorenzetti V Melo B Basilio R Suo C Yucel M Tierra-Criollo CJ Emotion regulation using virtual environments and real-time fMRI neurofeedback. Front Neurol (2018) 9:390. 10.3389/fneur.2018.00390 30087646 PMC6066986

[219] Gruzelier J EEG-neurofeedback for optimising performance. III: a review of methodological and theoretical considerations. Neurosci Biobehav Rev (2014) 44:159–82. 10.1016/j.neubiorev.2014.03.015 24690579

[220] Niv S Clinical efficacy and potential mechanisms of neurofeedback. Pers Individ Dif (2013) 54(6):676–86.

[221] Sulzer J Haller S Scharnowski F Weiskopf N Birbaumer N Blefari ML Real-time fMRI neurofeedback: progress and challenges. Neuroimage (2013) 76:386–99. 10.1016/j.neuroimage.2013.03.033 PMC4878436 23541800

[222] Carelli L Solca F Faini A Meriggi P Sangalli D Cipresso P Brain-Computer Interface for Clinical Purposes: Cognitive Assessment and Rehabilitation. BioMed Res Int (2017), 1695290. 10.1155/2017/1695290 28913349 PMC5587953

[223] Scott WC Kaiser D Othmer S Sideroff S Effects of an EEG biofeedback protocol on a mixed substance abusing population. Am J Drug Alcohol Abuse (2005) 31(3):455–69. 10.1081/ada-200056807 16161729

[224] Luigjes J Segrave R de Joode N Figee M Denys D Efficacy of invasive and non-invasive brain modulation interventions for addiction. Neuropsychol Rev (2019) 29(1):116–38. 10.1007/s11065-018-9393-5 PMC6499746 30536145

[225] Canterberry M Hanlon CA Hartwell KJ Li X Owens M Lematty T Sustained reduction of nicotine craving with real-time neurofeedback: exploring the role of severity of dependence. Nicotine Tobacco Res (2013) 15(12):2120–4. 10.1093/ntr/ntt122 PMC3819983 23935182

[226] Hanlon CA Hartwell KJ Canterberry M Li X Owens M LeMatty T Reduction of cue-induced craving through realtime neurofeedback in nicotine users: the role of region of interest selection and multiple visits. Psychiatry Res: Neuroimaging (2013) 213(1):79–81. 10.1016/j.pscychresns.2013.03.003 PMC4093788 23683344

[227] Hartwell KJ Hanlon CA Li X Borckardt JJ Canterberry M Prisciandaro JJ Individualized real-time fMRI neurofeedback to attenuate craving in nicotine-dependent smokers. J Psychiatry Neurosci (2016) 41(1):48. 26505139 10.1503/jpn.140200 PMC4688028

[228] Hartwell KJ Prisciandaro JJ Borckardt J Li X George MS Brady K Real-time fMRI in the treatment of nicotine dependence: a conceptual review and pilot studies. J Psychol Addictive Behav (2013) 27(2):501. 10.1037/a0028215 PMC3646943 22564200

[229] Li X Hartwell KJ Borckardt J Prisciandaro JJ Saladin ME Morgan PS Volitional reduction of anterior cingulate cortex activity produces decreased cue craving in smoking cessation: a preliminary real-time fMRI study. Addict Biol (2013) 18(4):739–48. 10.1111/j.1369-1600.2012.00449.x PMC3389595 22458676

[230] Kim D-Y Yoo S-S Tegethoff M Meinlschmidt G Lee J-H The inclusion of functional connectivity information into fMRI-based neurofeedback improves its efficacy in the reduction of cigarette cravings. J Cog Neurosci (2015) 27(8):1552–72. 10.1162/jocn_a_00802 25761006

[231] Karch S Keeser D Hümmer S Paolini M Kirsch V Karali T Modulation of craving related brain responses using real-time fMRI in patients with alcohol use disorder. PloS One (2015) 10(7):e0133034. 26204262 10.1371/journal.pone.0133034 PMC4512680

[232] Kirsch M Gruber I Ruf M Kiefer F Kirsch P Real-time functional magnetic resonance imaging neurofeedback can reduce striatal cue-reactivity to alcohol stimuli. Addict Biol (2016) 21(4):982–92. 10.1111/adb.12278 26096546

[233] Ekhtiari H Tavakoli H Addolorato G Baeken C Bonci A Campanella S Transcranial electrical and magnetic stimulation (tES and TMS) for addiction medicine: A consensus paper on the present state of the science and the road ahead. Neurosci Biobehav Rev (2019) 104:118–40. 10.1016/j.neubiorev.2019.06.007 PMC7293143 31271802

